# Cytotoxic stress induces transfer of mitochondria-associated human endogenous retroviral RNA and proteins between cancer cells

**DOI:** 10.18632/oncotarget.21606

**Published:** 2017-10-07

**Authors:** David Díaz-Carballo, Jacqueline Klein, Ali H. Acikelli, Camilla Wilk, Sahitya Saka, Holger Jastrow, Gunther Wennemuth, Phillip Dammann, Urs Giger-Pabst, Veria Khosrawipour, Joachim Rassow, Mikalai Nienen, Dirk Strumberg

**Affiliations:** ^1^ Institute for Molecular Oncology and Experimental Therapeutics, Department of Hematology and Medical Oncology, Marienhospital Herne, Ruhr-University of Bochum, Bochum, Germany; ^2^ Institute of Anatomy and Experimental Morphology, University of Duisburg-Essen, Essen, Germany; ^3^ Central Animal Laboratory, University of Duisburg-Essen, Essen, Germany; ^4^ Department of Surgery, Marienhospital Herne, Ruhr-University of Bochum, Bochum, Germany; ^5^ Institute of Biochemistry and Pathobiochemistry, Department of Cellular Biochemistry, Ruhr-University of Bochum, Bochum, Germany; ^6^ Department of Nephrology, Marienhospital Herne, Ruhr-University of Bochum, Bochum, Germany

**Keywords:** human endogenous retroviruses (HERVs), mitochondria, intercellular mitochondrial transfer, syncytin-1/2 receptors

## Abstract

About 8 % of the human genome consists of human endogenous retroviruses (HERVs), which are relicts of ancient exogenous retroviral infections incurred during evolution. Although the majority of HERVs have functional gene defects or epigenetic modifications, many of them are still able to produce retroviral proteins that have been proposed to be involved in cellular transformation and cancer development.

We found that, in chemo-resistant U87^RETO^ glioblastoma cells, cytotoxic stress induced by etoposide promotes accumulation and large-scale fission of mitochondria, associated with the detection of HERV-WE_1_ (syncytin-1) and HERV-FRD_1_ (syncytin-2) in these organelles. In addition, mitochondrial preparations also contained the corresponding receptors, i.e. ASCT2 and MFSD2. We clearly demonstrated that mitochondria associated with HERV-proteins were shuttled between adjacent cancer cells not only via tunneling tubes, but also by direct cellular uptake across the cell membrane. Furthermore, anti-syncytin-1 and anti-syncytin-2 antibodies were able to specifically block this direct cellular uptake of mitochondria even more than antibodies targeting the cognate receptors.

Here, we suggest that the association of mitochondria with syncytin-1/syncytin-2 together with their respective receptors could represent a novel mechanism of cell-to-cell transfer. In chemotherapy-refractory cancer cells, this might open up attractive avenues to novel mitochondria-targeting therapies.

## INTRODUCTION

The human genome contains several copies of human endogenous retroviruses or HERVs, constituting about 8 % of the genetic material [1–8]. HERV elements are identified according to their retroviral genetic hallmarks such as the *gag*, *pol* and *env* genes flanked by non-coding long terminal repeats (LTRs). HERVs are categorized into three classes based on exoviral sequence homologies: Class I, broadly clustering with ε (epsilon) and γ (gamma) viruses, Class II, clustering with β (beta) viruses, and Class III, the members of which are most closely related to spumaviruses. The individual subclasses are defined by the predicted tRNA specificity of the binding site at which reverse transcription will be initiated [1–5]. In contrast to their retroviral ancestors and murine or porcine counterparts, HERVs have not been reported to generate infectious viral particles in humans. Due to mutations and epigenetic modifications, they have lost the capacity of horizontal transmission and are merely inherited as a part of the genome. However, most of their LTRs have retained functional promotors, and therefore many HERVs do contain protein-encoding genes [6–10]. In fact, some of these proteins are known to have physiological functions, while others appear to be synthesized only in pathological conditions [11–14]. For instance, the envelope protein from multiple sclerosis (MS) associated retroviral element (MSARV), a member of the HERV-type W, induces impaired immunity and promotes inflammation [15].

Furthermore, most cancer cells show atypical gene expression patterns, often involving epigenetic modifications [16]. There is increasing evidence that these mechanisms may also affect the expression of HERV proteins [12, 14]. Enhanced expression of specific HERV proteins has been described to occur in different tumors, including HERV-K (HML6) in melanoma, HERV-K (HML2) in germ-cell carcinoma, and HERV-E in renal cell carcinoma [8, 17–20]. Augmented expression of syncytin 1 was observed in cells from different malignancies [20–22]. Little is known about the biochemical activities of the specific HERV-proteins found in tumors. However, some of them seem to contribute to cancer development and some mechanisms of action have been proposed [6, 7, 17, 20]. For instance, HERV-K expression is correlated with the prognosis and progress of hepatocellular carcinoma [23]. HERV-K activation is strictly required to sustain CD133^+^ melanoma cells with stemness features [17]. Recently, it was reported that activation of HERV-K env protein is essential for tumorigenesis and metastasis formation of breast cancer cells [24]. Furthermore, we recently found that enhanced HERV-expression is associated with the development of chemo-resistance in colon carcinoma cells [25].

Tumor cells have many interactions with surrounding malignant and non-malignant cells which are recruited to the tumor site. Some of these interactions are essential to tumor growth and metastatic spread [26–28]. Direct intercellular contact via tunneling nanotubes has recently been shown to support the cell-to-cell transfer of cytosolic molecules and even intact organelles [29–31]. It was also reported that intercellular exchange of mitochondria occurs between different cells, including cancer cells and endothelial cells, which may have a modulating effect on chemo-resistance. In agreement with this, we noted a highly chemo-resistant cancer cell population showing intense mitochondrial traffic between cells. Furthermore, it was recently reported that high mitochondrial mass betrays a sub-population of stem-like cancer cells that are chemo-resistant [32].

Apart from cell-to-cell transfer via tunneling nanotubes, vesicle transfer and cell-cell fusion are emerging novel mechanisms for modulating cancer cells. This cellular fusion process is strictly regulated by proteins that carry the information to organize and regulate membranes into merging two separate lipid bilayers into one [33].

HERV molecules have not been linked to mitochondria until now. Here, we suggest that HERV proteins are not inertly exchanged amongst mitochondria. The HERV envelope-proteins WE_1_ (syncytin-1) and FRD_1_ (syncytin-2) appear to be highly affine to mitochondria, and may even facilitate their intercellular exchange via free uptake across the cell membranes. In support of this hypothesis, anti-syncytin-1 and anti-syncytin-2 antibodies were able to block cellular uptake of isolated mitochondria.

The results of our studies underpin the assumption that the fusogenic properties of HERV envelope proteins syncytin-1 and syncytin-2 are required and sufficient to enable mitochondrial cell-to-cell transfer among chemotherapy-refractory cancer cells. This novel cellular mechanism of syncytin-mediated mitochondrial transfer could play a role in conferring resistance to anti-cancer therapy and might provide attractive avenues to new mitochondria-targeted therapies.

## RESULTS

### Cytotoxic stress induces perinuclear accumulation of mitochondria liable to intercellular exchange via tunneling

**Figure 1:**
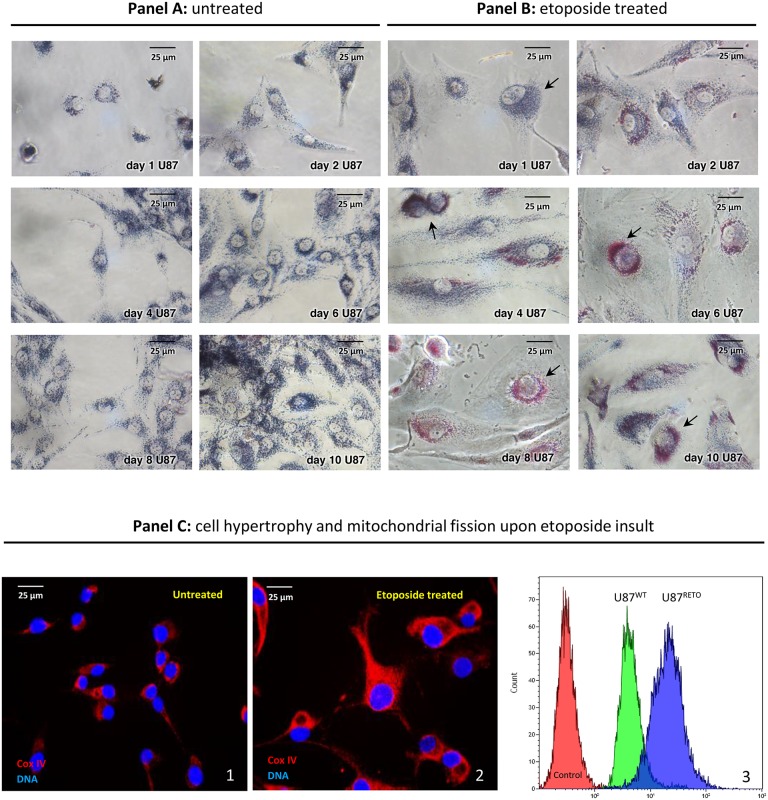
Mitochondrial accumulation in the perinuclear area in U87^RETO^ glioblastoma cells upon stress with etoposide Panel **A1-10** days: MTT staining, untreated control of U87^WT^ glioblastoma cells. After 10 days, cells were incubated with 1mg/ml MTT for two hours for mitochondrial visualizing (blue). Panel **B1-10** days: visualization of mitochondrial accumulation upon etoposide incubation at 5 μg/ml final concentration for a total of 10 days in U87^RETO^ glioblastoma cells. Perinuclear rearrangement of mitochondria was already seen after 2 days of drug incubation (i.e. day 2 of panel B) and increased during time exposure. Cells depict hypertrophic bodies in response to etoposide stress. Panel **C:** semi-quantification of mitochondrial fission upon stress with etoposide. Panel **C1** (untreated) depicts the mitochondrial distribution by visualizing the expression of Cox IV in U87^WT^ cells. Panel **C2** (etoposide treated) represents the distribution of mitochondria in U87^RETO^ cells after 10 days of drug exposure as visualized by Cox IV fluorescent detection. Treated cells show hypertrophy and massive perinuclear rearrangement of mitochondria, compared to untreated cells. Panel **C3** presents flow cytometric analysis of both U87^WT^ and U87^RETO^ cells labeled with red MitoTracker^®^. Control: Untreated cells. U87^WT^ and U87^RETO^ cells treated with etoposide as described above. Enhanced mitochondrial accumulation could be confirmed. Magnification 40x. The figure is representative of at least n = 3 independent experiments.

U87 glioblastoma cells showed a remarkable mitochondrial aggregation around the nucleus in response to 5 μg/ml of the cytotoxic drug etoposide, a specific topoisomerase II inhibitor, [24, 25] (Figure 1, panel **B** 1-10 days). The resulting chemo-resistant subline obtained by etoposide exposure, U87^RETO^, expressed stem cell features, compared to the original wild-type line U87 [25, 32]. Upon etoposide exposure, perinuclear mitochondrial accumulation became evident already after 2 days (i.e. day 2 in panel B) and further increased during prolonged drug exposure. Untreated U87-control cells showed proliferation without detectable changes in cellular morphology or subcellular mitochondrial distribution (Figure 1, panel A 1-10, days). In contrast, etoposide-treated cells in panel B displayed reactive cell hypertrophy and remarkable perinuclear mitochondrial concentration.

To confirm this observation, we also performed Cox IV staining on untreated (panel **C1**) and etoposide-treated (5 μg/ml final concentration for a total of 10 days, panel **C2**) U87 cells. Again, the treated cells showed massive perinuclear accumulation of mitochondria in comparison with untreated cells.

Panel **C3** further confirms enhanced mitochondrial accumulation in both U87^WT^ and U87^RETO^ cells upon etoposide treatment. Although already detectable in U87^WT^, this effect was even more conspicuous in resistant U87^RETO^ cells.

Our results are consistent with previous reports on a sub-population of stem-like breast cancer cells, showing that increased mitochondrial mass may be associated with chemoresistance [34].

**Figure 2:**
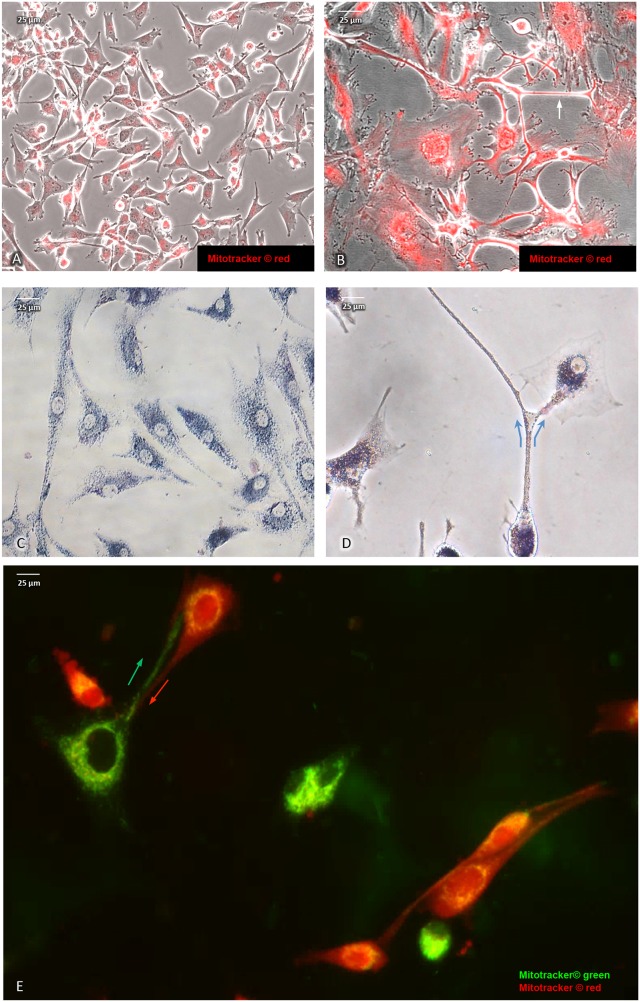
Intercellular exchange of mitochondria via nano-tubes in U87^RETO^ glioblastoma cells **A** and **B**: mitochondria labeled with red MitoTracker^®^, picture A: untreated control, picture B: same cells in response to etoposide added at 5 μg/ml final concentration for a total of 10 days. Pictures **B** and **C**: intercellular exchange of mitochondria via nano-tubes as observed by MTT and red MitoTracker^®^ staining (see arrows). Pictures **A** and **C:** untreated controls. Picture **E:** co-cultivation of mitochondria of U87^RETO^ cells previously labeled with MitoTracker^®^ red and green. An exchange of mitochondria via nano-tubes as well as perinuclear arrangement of newly acquired mitochondria around host nucleus are clearly visible (see arrows).

Direct intercellular contact by tunneling nanotubes has recently been reported to support cell-to-cell transfer of cytosolic molecules and mitochondria also among cancer cells [29]. In addition, upon cytotoxic stress induced by etoposide (5 μg/ml final concentration for a total of 10 days), we noted an intercellular exchange of mitochondria via tunneling (Figure 2B & 2D) in U87^RETO^ cells that was less pronounced in untreated controls (panel **A** & **C**). Figure 2E shows U87^RETO^ cells with either “red” or “green” MitoTracker™-labeled mitochondria. These populations were then mixed while being incubated with etoposide (5 μg/ml final concentration for a total of 10 days). This experiment confirmed the exchange of labeled mitochondria among adjacent cells via nanotubes upon cytotoxic stress (Figure 2E, arrows) with subsequent perinuclear concentration of newly acquired mitochondria.

We also reported that chemotherapy resistance was associated with enhanced HERV expression in tumor cells with stem cell phenotypes [25]. We next investigated whether HERV overexpression is associated with the observed perinuclear rearrangement of mitochondria.

### Stress-induced association of mitochondria with HERV-envelope proteins

**Figure 3:**
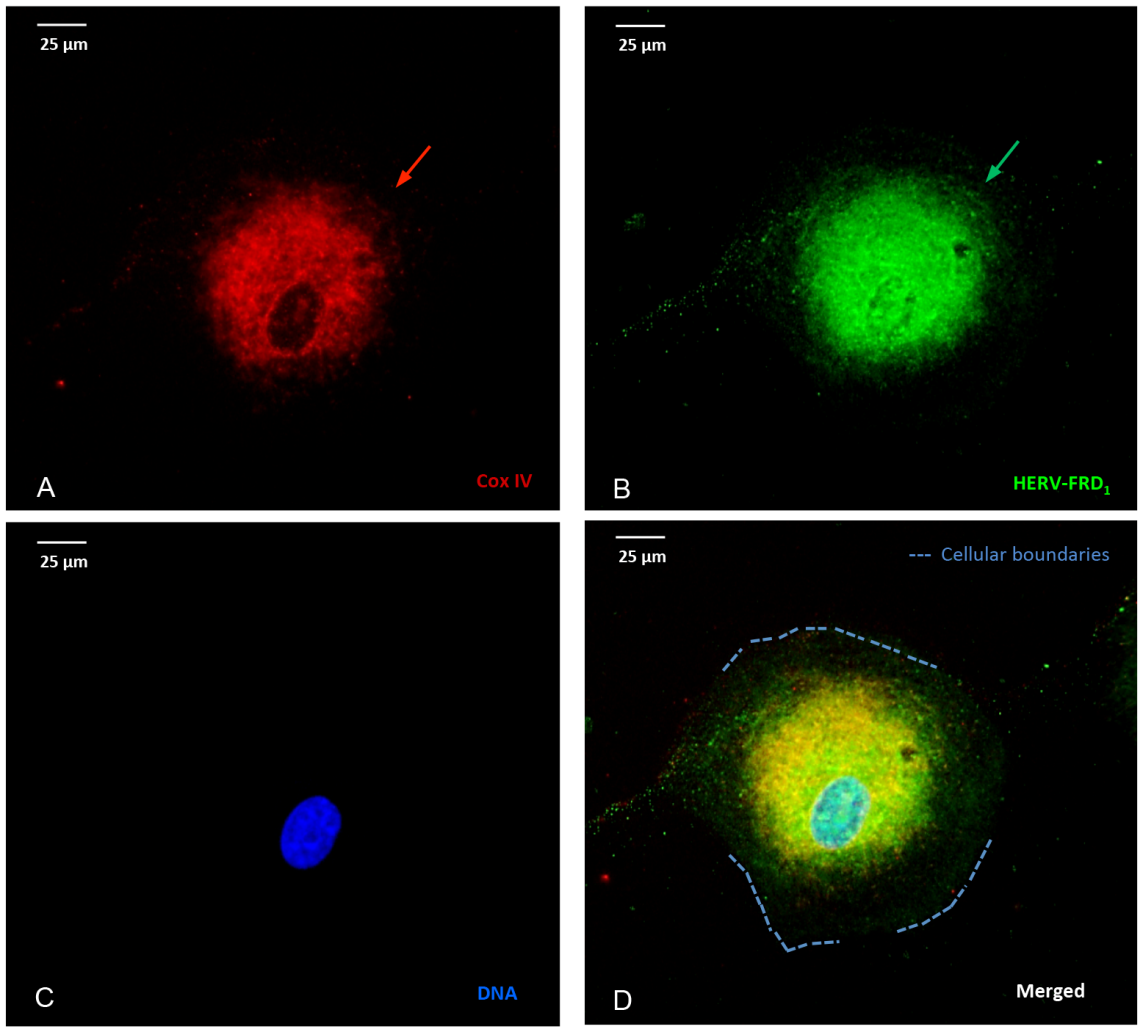
Stress-induced, co-localization of mitochondria and HERV-proteins Cellular localization of HERV proteins in U87^RETO^ cells upon etoposide exposure at 5 μg/ml final concentration (for 10 days) measured by immunocytochemistry. The intracellular distribution of syncytin 2 (HERV- FRD_1_) was associated with mitochondria. Picture **A**: Cox IV staining, picture **B**: staining of HERV- protein using anti-syncytin 2-FITC conjugated antibody. Same results were obtained using anti-HERV-FITC conjugated antibodies against HERV-V_3-1_ and syncytin 1 (HERV-W_E1_), respectively (not shown). Picture **C**: negative control, DNA was labeled with DAPI, picture **D**: combined staining with all three merged channels. Magnification 40x. The figure is representative of at least n = 3 independent experiments.

Figure 3 depicts the intracellular distribution of HERV- proteins in U87^RETO^ cells after incubation with 5 μg/ml etoposide (Figure 3A-3D). HERV-FRD_1_ proteins appear to be predominantly associated with mitochondria (**3A**) and co-localized to the perinuclear space (**3B**, arrows). This was also observed for various HERV elements such as HERV- W_E1_ (syncytin 1) and HERV-V_3-1_ (data not shown).

### Molecular analysis of HERV proteins in isolated mitochondria

**Figure 4:**
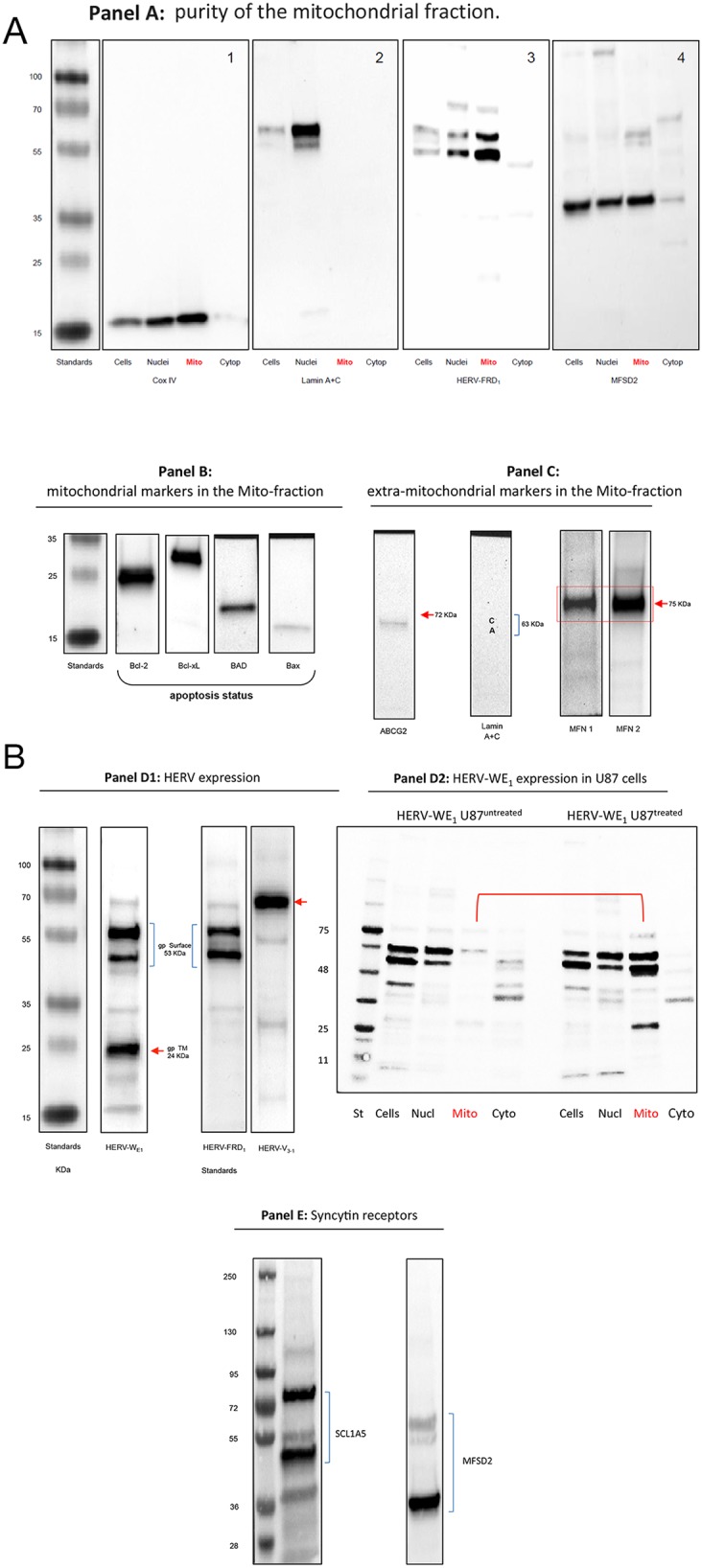
Western blot analysis of HERV-proteins in the mitochondrial fraction of U87^RETO^ glioblastoma cells upon etoposide incubation with 5 μg/ml for 10 days Panel **A:** controls. **A1**: Mitochondrial Cox IV was detected in whole cell extracts, nuclear and mitochondrial fractions. **A2**: lamin A & C markers were detected only in nuclear fractions indicating the absence of nuclear contaminations in the mitochondrial fraction except for cytoplasmic preparations. Panel **A3** reflects the content of syncytin 2 (HERV-FRD_1_) in all sub-cellular preparations with remarkable expression in the mitochondrial fraction of U87^RETO^ cells. Panel **A4**: detection of the receptor for syncytin 2, MFSD2 in subcellular fractions except for cytoplasmic preparations. Panel **B** minor expression of pro-apoptotic proteins like BAD and BAX in U87^RETO^ cells. In contrast, anti-apoptotic proteins like Bcl-2 and Bcl-xL were found strongly expressed. (For comparison of cytoplasmic vs. whole cell distributions of BAX in untreated vs. treated cells, showing no difference upon etoposide-incubation, see Supplementary Data.) Panel **C**: controls: isolated mitochondria with mitochondrial markers MFN1 and MFN2 (positive control) almost without extra-mitochondrial membrane proteins like ABCG2 and lamin A+C (negative controls). Panel **D1**: expression of different HERV proteins in the mitochondrial fraction of U87^RETO^ cells. For syncytin 1 (HERV-W_E1_, upper bands) the 53 kDa surface protein and an additional splicing variant below is shown. In addition, the 24 kDa band reflects the transmembrane protein of syncytin 1. For syncytin 2 the 24 kDa protein was not detectable. HERV-V_3-1_ was detectable as the expected single band at 32 kDa. Panel **D2**: analysis of intracellular distribution of syncytin 1 in untreated U87^RETO^ cells (control, left lanes) vs. treated U87^RETO^ cells after incubation with 5 μg/ml etoposide for 10 days. HERV proteins were found enhanced in the mitochondrial fraction upon etoposide stress. Comparable results were obtained for syncytin 2, see Supplementary Data. Panel **E**: after incubating U87^RETO^ cells with 5 μg/ml etoposide for 10 days, receptors for syncytin 1 and 2 were also clearly detectable in mitochondrial fraction (see also panel **A4**). For SCL1A5, the endogenous protein (53 kDa) and various glycosylated forms were detected. The receptor for syncytin 2, MFSD2, was also detectable as a single band. The figure is representative of at least n = 3 independent experiments.

To further explore a possible association of HERV proteins and mitochondria, we examined protein extracts from isolated mitochondria using Western blotting. Figure 4A-4D1, 4E shows mitochondrial proteins extracted from U87^RETO^ glioblastoma cells after incubation with 5 μg/ml etoposide.

Serving as a control, panel **A** shows that the mitochondrial marker Cox IV is present in whole cell preparations as well as in nuclei and mitochondria, but not in isolated cytoplasm (panel **A1**). In addition, lamin A+C was mainly detected in the nuclear fraction (panel **A2**). This analysis indicates that the mitochondrial fraction was not contaminated with nuclear proteins. Figure 4, panels **A 3** & **4** depict the distribution of syncytin 2 and its receptor MFSD2 in the subcellular preparations.

### Physiological status of the isolated mitochondria

Some viral proteins are known to interact with mitochondrial structures and thereby affect apoptosis. Viruses may hijack or mimic mitochondrial proteins, the latter by encoding homologs of the anti-apoptotic Bcl-2 [35–40]. They can deplete or degrade mitochondrial DNA and modulate the mitochondria-mediated antiviral immunity mechanism, for example by abrogating the production of interferon that mediates the cellular response to viral invasion [41, 42].

The prolonged incubation with the cytotoxic agent etoposide suggests that at least a considerable amount of cells might undergo apoptosis. Figure 4, panel **B** shows only minimal quantities of pro-apoptotic proteins like BAD and BAX. In contrast, anti-apoptotic proteins like Bcl-2 and Bcl-xL are overexpressed. BAX has been reported to be transported to the mitochondria from the cytoplasm upon apoptotic stimulation. We therefore analyzed whole cell/cytoplasmic distributions of BAX in untreated versus treated U87 cells. No substantial difference was established upon etoposide incubation (see Supplementary Data). Further confirmation that these etoposide-treated cells are not apoptotic was provided by the absence of caspase-3 cleavage (data not shown).

Taken together, our data indicate that the cells exposed to etoposide are not in an apoptotic state. However, it remains to be elucidated whether HERV proteins do have any impact on apoptosis or inhibit the mitochondrial apoptotic mechanism.

Figure 4, panel **C** reflects additional controls included for the mitochondrial preparations. Western blot analysis clearly reveals the presence of mitochondrial markers such as MFN1 and MFN2 (mitofusins) but almost no extra-mitochondrial membrane proteins like ABCG2 and lamin A+C.

At least three different HERV envelope proteins were detected in the mitochondrial fraction (Figure 4, panel **D1**). For the syncytin 1 equivalent HERV-W_E1_, the upper bands represent the 53 kDa surface subunit (2 bands detected with the commercially available antibody, which reflects splicing variants) and the 24 kDa transmembrane subunit of HERV-W_E1_ below, which was previously reported by Cheynet et al. [43]. Comparable results were obtained for syncytin 2 which is HERV-FRD_1_, except for the 24 kDa transmembrane subunit. For the HERV-V_3-1_ *env* protein, a single band at 68 kDa was detected.

Figure 4, panel **D2** shows that syncytin 1 in untreated U87 cells (control, left lanes) is predominantly localized in the whole-cell lysates and nuclear fractions, with only small amounts detectable in the mitochondrial and cytoplasmic fractions. However, upon cytotoxic stress with 5 μg/ml etoposide over 10 days, HERV protein expression appear to be increased in the mitochondrial fraction, indicating a translocation of HERVs to the mitochondria. Comparable results were obtained for HERV-FRD_1_ (see Supplementary Data).

Figure 4, panels **A4** & **E** show that, in mitochondria from U87^RETO^ cells after cytotoxic stress induced with etoposide, both syncytin 1 and 2 and the corresponding receptors are detectable. For SCL1A5, we show the endogenous protein (53 kDa) and various glycosylated forms. The receptor for syncytin 2, MFSD2, is detectable as a strong signal at 40 KDa. In addition, as control, neither HERV-proteins nor specific syncytin receptors were detectable in the cytoplasm-fraction of analyzed cells (Figure 4, panel **A 3** & **4**). For technical reasons, the nuclear preparation was contaminated with mitochondria and mitochondria-associated HERV-proteins.

These data clearly indicate that mitochondria isolated from etoposide-stressed cancer cells might be subjected to syncytin 1 and 2 mediated membrane fusion, because major fusogenic HERV envelope proteins and their specific receptors were identified in the same preparation.

**Figure 5:**
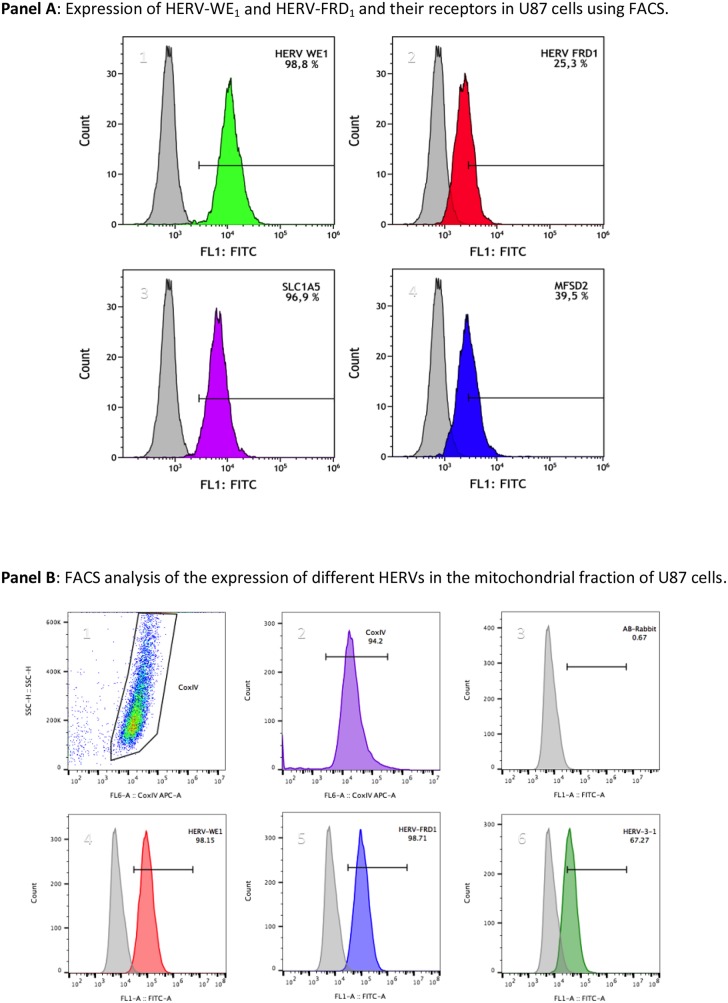
Flow cytometric analyses of the localization of HERVs in the mitochondrial fraction of U87^RETO^ glioblastoma cells upon incubation with etoposide Mitochondrial samples were dual labeled for Cox IV and different HERV-proteins. Panel **A** depicts the expression of syncytin 1 (HERV-WE_1,_ **A1**), syncytin 2 (HERV-FRD_1_, **A2**), their respective receptors SLC1A5 **(A3)** and MFSD2 **(A4)** in U87 cells, which were used as source of mitochondria. The total HERV-protein expression in whole U87 cells was set to 100 %, and the relative amount of HERV-proteins associated with the mitochondrial fraction was given in relationship to the total HERV-protein amount in whole cells. Panel **B** depicts the measurement of different HERVs in the mitochondrial fraction of these U87 glioblastoma cells. The Cox IV- positive gated populations represent more than 94 % (picture **B2**). Picture **B3** represents the negative control (secondary antibody). Both syncytin 1 and syncytin 2 (**B4** & **B5**) were found to be highly expressed, accounting for more than 98 %. HERV-V_3-1_ (**B6**) was detected in more than 67 % of the Cox IV positive populations. Plots are representative of at least n = 3 experiments.

To confirm that mitochondria carry HERV-proteins, we also performed fluorescence flow cytometry. Figure 5, panel **A** reflects the detection of syncytin 1 and syncytin 2 and their respective receptors SLCA5 and MFSD2 in U87 cells.

Mitochondria isolated from U87^RETO^ were analyzed for the expression of different HERVs. Cox IV-positive populations were gated (Figure 5, panels **B 1-2**) to fix the population to be analyzed, which corresponds to the mitochondria (> 94 %). Panel **A3** shows the negative control (secondary antibody). Figure 5, panels **B 4-6** depict the histograms of different HERV envelope proteins which co-segregate with mitochondria for more than 98 % positivity for syncytin 1, syncytin 2 and for approximately 67 % for HERV-V_3-1_. Following this additional approach, we distinguished the presence of HERV proteins in the mitochondrial fraction.

### Isolated mitochondria from chemo-resistant U87^RETO^ cells carry various HERV-RNA transcripts

**Figure 6:**
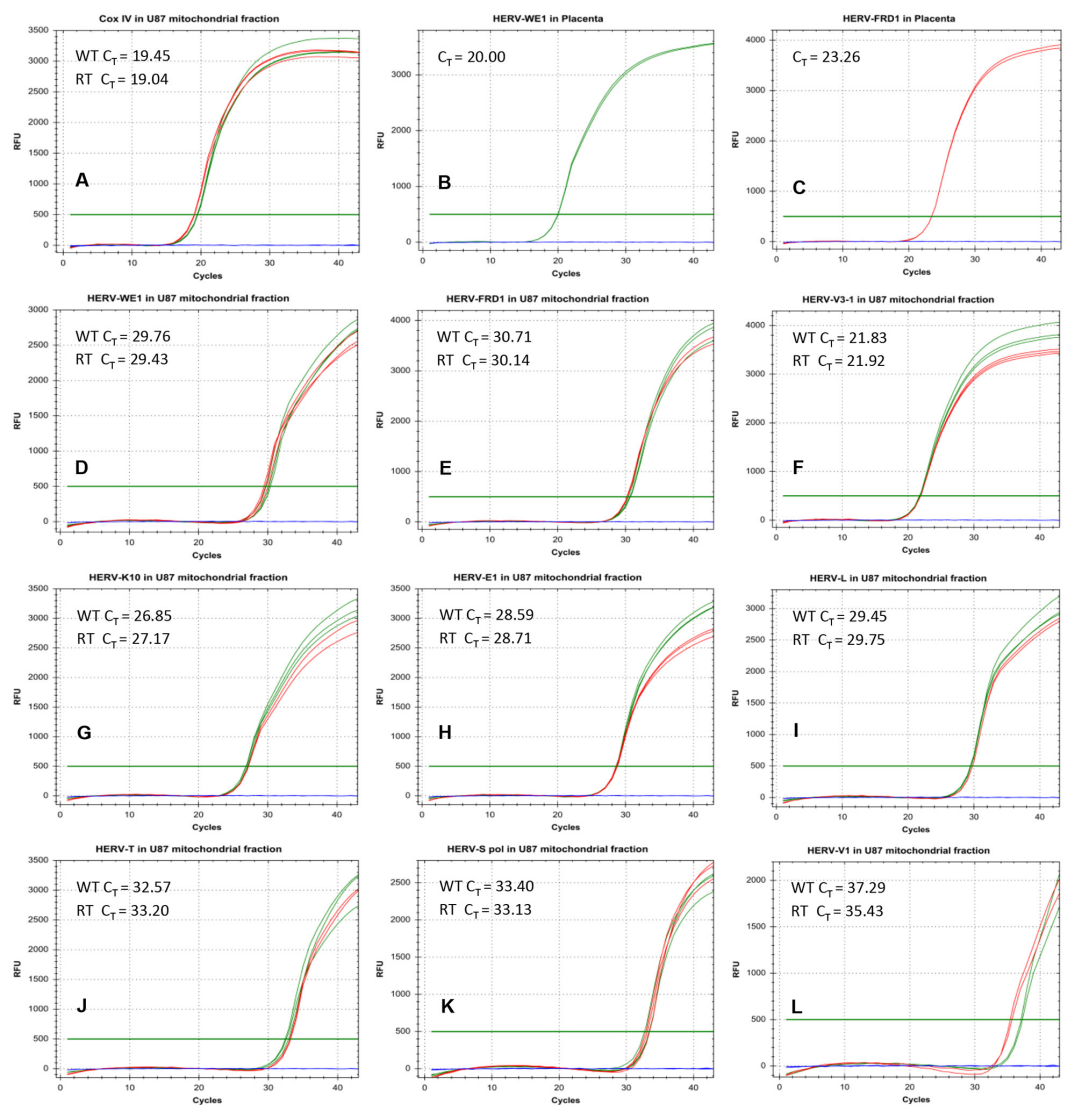
Real-time PCR analysis of HERV-RNA transcripts in the mitochondrial fraction of U87^RETO^ glioblastoma cells Figures **A-C** shows various positive controls (Cox IV in mitochondria and syncytin 1 (HERV-WE_1_) as well as syncytin 2 (HERV-FRD_1_) in placenta tissues). In general, we defined a C_T <_ 30 as a standard for positive RNA amplification. This threshold was achieved for CoxIV in mitochondria (picture A), and for syncytin 1 (picture B) and syncytin 2 (panel C) in placenta tissues. In pictures **D-L**, various HERV-transcripts were analyzed in the mitochondrial fraction. Only for HERV-V_3-1_ and HERV-K_10_, the standard for positive transcriptional activity (C_T_ = 21.8) was reached (pictures F and G). Green and red curves represent the RNA from the mitochondrial fraction of untreated and treated cells, respectively. Results were shown for at least 5 independent experiments.

Since various HERV envelope proteins were identified in mitochondria, we next addressed the question whether these organelles also carry RNA coding for these proteins. We therefore performed real-time PCR experiments to detect HERV transcripts using specific TaqMan primers and probes (Figure 6).

Figure 6A-6C shows various positive controls. In general, we defined a C_T_ of< 30 cycles as a limit for positivity applying 100 ng of cDNA. In panels **D-L**, various HERV-transcripts were analyzed in the mitochondrial fraction. Substantial amounts of HERV-RNAs were only detectable for HERV-V_3-1_ (**6F**) and HERV-K_10_ (**6G**). For other HERVs analyzed, although detectable, their quantities were much lower (Figure 6D, 6E, 6I, 6J, 6K & 6L). This confirms that mitochondria carry not only HERV-proteins, but also potentially HERV-RNA, as shown for HERV-V_3-1_ and HERV-K_10_. Green curves represent RNA from untreated cells and red curves from etoposide treated cells, showing that the amount of RNA was not affected by etoposide treatment.

### Direct trans-membrane uptake of purified and labeled mitochondria by cancer cells

**Figure 7:**
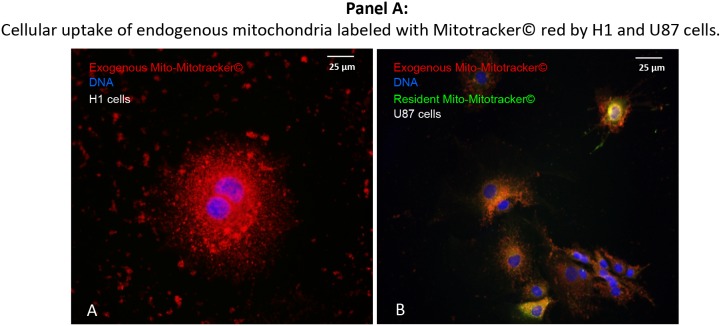
Direct, trans-membranous cellular uptake of purified and labeled mitochondria by cancer cells Picture **A**: the donor-mitochondria were previously isolated from U87^RETO^ cells and labeled with red MitoTracker^®^. These prepared “red” mitochondria were added to H1^RETO^ testicular carcinoma cells, whose endogenous mitochondria were not labeled. This effect was also detectable in wild-type U87 cells (data not shown). Picture **B**: the same prepared “red” mitochondria were added to U87^RETO^ glioblastoma cells, whose endogenous mitochondria were previously labeled with green MitoTracker^®^. Incorporation was measured for at least 24 h. DNA was labeled with DAPI. Magnification 20x. The figure is representative of at least n = 3 independent experiments.

In a next step, we analyzed whether cancer cells of different histology are capable of taking up purified exogenous mitochondria in the absence of pre-existing nanotubes (Figure 7). For this purpose, isolated donor-mitochondria were labeled with red MitoTracker^®^. These “red” mitochondria were then added to H1^RETO^ testicular carcinoma cells, whose endogenous mitochondria were unlabeled (Figure 7A). It was found that labeled “red” mitochondria reached the cytoplasm of H1^RETO^ cells without detectable tunneling nanotubes. This effect was also seen in wild-type U87 cells (data not shown). Next, we added “red” labeled mitochondria to U87^RETO^ cells, which served as recipient containing endogenous mitochondria previously labeled with “green” MitoTracker^®^. After co-cultivation for 24 hours without cytotoxic stress, cellular uptake of “red” mitochondria by U87^RETO^ cells was documented. Figure 7B clearly shows that U87^RETO^ cells took up the exogenous mitochondria without preexisting tunneling nanotubes, indicating that U87^RETO^ cells contain a mix of endogenous “green” mitochondria and exogenous “red” mitochondria in the cytoplasm.

### Uptake of purified mitochondria carrying HERV-proteins including syncytin 1 by cancer cells

**Figure 8:**
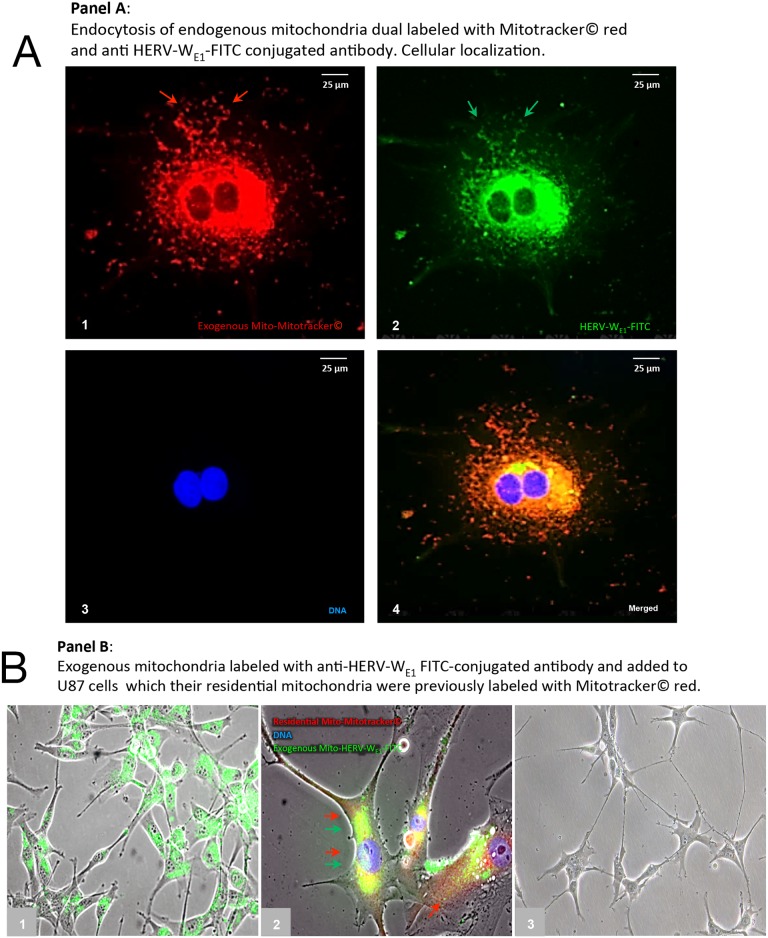
Cellular uptake of purified mitochondria carrying syncytin 1 by cancer cells Panel **A**: trans-membranouscellular uptakeof prepared mitochondria, which were previously dual labeled with red MitoTracker^®^ (picture **A1**) and simultaneously with anti-syncytin 1 (HERV-W_E1_) antibody-FITC (picture **A2**) into U87^RETO^ cells (endogenous mitochondria notlabeled). Picture **A3**: negative control, DNA was labeled with DAPI. Picture **A4**: combined staining with all three merged channels. Magnification 40x. The figure is representative of at least n = 3 independent experiments. Panel **B**, picture **B1**: cellular uptakeof prepared mitochondria, which were previously labeled only with anti-syncytin 1 antibody-FITC into U87^RETO^ cells, with endogenous mitochondria previously not labeled. Picture **B2**: trans-membranous **c**ellular uptake of prepared mitochondria, which were previously labeled only with anti-syncytin 1 antibody-FITC into U87^RETO^ cells, with endogenous mitochondria previously labeled with red MitoTracker^®^. Picture **B3:** negative control: endogenous mitochondria not labeled and prepared mitochondria previously only labeled with rabbit-FITC conjugated secondary antibody.

We next sought to investigate whether direct cellular uptake of mitochondria is associated with uptake of HERV-proteins labeling the mitochondrial fraction isolated from U87^RETO^ with anti-syncytin 1 antibody-FITC conjugated. For this purpose, mitochondria labelled with both red MitoTracker^®^ and anti-syncytin 1antibody-FITC were added to H1^RETO^ testicular carcinoma cells. After 24 h incubation without further cytotoxic stress, massive incorporation of HERV-carrying mitochondria into H1^RETO^ cells was noted (Figure 8, panels **A1-4**). Since neither tunneling nanotubes nor other membrane-bound cell-bridging structures from adjacent cells were present, we conclude that a direct trans-membrane uptake of these prepared mitochondria into the cancer cells took place. Moreover, mitochondria from different cells were found to be interchangeable.

We next aimed to confirm a possible trans-membrane uptake of prepared mitochondria from U87^RETO^ that were previously labeled with anti-syncytin 1-FITC (Figure 8, panel **B1**) or labeled with both anti-syncytin 1-FITC and red MitoTracker^®^ (Figure 8, panel **B2**). These preparations were then added to U87^WT^ cells. Again, after 24 h incubation, an uptake of exogenous mitochondria was observed. Figure 8, panel **B3** shows untreated U87^WT^ as control.

In summary, we have established the incorporation of isolated mitochondria carrying HERV proteins into cells as a mechanism of direct trans-membrane uptake of these prepared mitochondria into cancer cells regardless of histological origin.

### Blocking the cellular uptake of purified mitochondria with anti-syncytin 1 or anti-syncytin 2 antibodies

**Figure 9:**
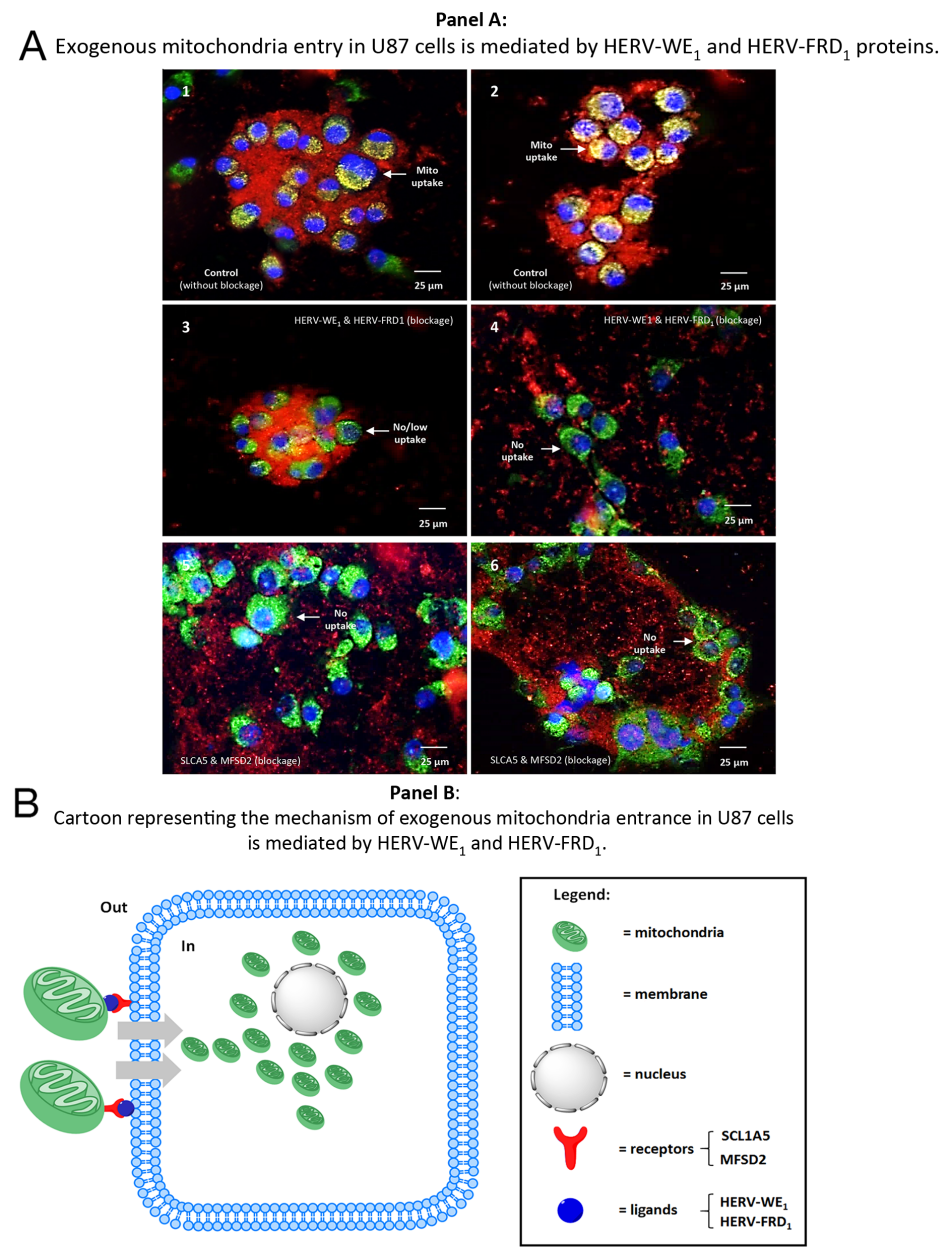
Blocking the cellular uptake of purified mitochondria using antibodies against syncytin 1 or syncytin 2 Panel **A 1** & **2**: positive controls, mitochondria were previously isolated from U87^RETO^ cells after cytotoxic stress with etoposide (as described above) and labeled with red MitoTracker^®^. Endogenous mitochondria of the U87 host-cells were stained with green MitoTracker^®^. After 24h-incubation without further cytotoxic stress, cellular uptake of prepared “red” mitochondria into cancer cells was clearly detectable. Panels **3-6**: experiments were performed under the same conditions as described for panels **A 1** & **2**. Panels **A 3** & **4**: mitochondria isolated from U87^RETO^ were blocked with both anti-syncytin 1 (HERV-W_E1_) and anti-syncytin 2 (HERV-FRD_1_) blocking antibodies and the uptake of the labeled mitochondria was monitored after 24 hours. Panels **A 5** & **6**: mitochondria isolated from U87^RETO^ were blocked with both anti-SLC1A5 and anti-MFSD2 blocking antibodies and the uptake of the labeled mitochondria was monitored after 24 hours. These results indicate that syncytin 1 and 2 as well as their receptors play a substantial role in cellular uptake of mitochondria, since the uptake of exogenous mitochondria were less perceived after blockage of these proteins. Panel **B**: cartoon representation of the proposed mechanisms of mitochondria entrance into U87 cells. Mitochondria from U87^RETO^ etoposide resistant cells were isolated and labeled with MitoTracker© red and with anti syncytin 1 or anti syncytin 2 antibodies and exogenously applied to U87 cells. The blockage of both syncytins impedes the uptake of free mitochondria.

So far, we have presented evidence for the direct cellular uptake of mitochondria carrying HERV-proteins, i.e. syncytin1 and 2 and their cognate receptors. First, as a control we monitored the uptake of mitochondria in the absence of blocking antibodies. For this purpose, we labeled resident mitochondria of U87^WT^ cells with green MitoTracker^®^. Exogenous mitochondria from U87^RETO^ cells were labeled with red MitoTracker^®^ and then added to the U87^WT^ cells (Figure 9, panels **A1-2**). We demonstrate the uptake of exogenous mitochondria across the cell membrane. Secondly, to substantiate that the uptake of mitochondria by cancer cells is mediated by these HERV proteins and their cognate receptors, we blocked these proteins using specific antibodies (anti-syncytin 1, anti-syncytin 2, Figure 9, panels **A3-4**, or anti-SLCA5 and anti-MFSD2, Figure 9, panels **A5-6**). The blocked mitochondria were found to be almost completely unable to cross the cellular membrane. However, as syncytin 1 and syncytin 2 are highly similar to the envelope proteins from other endogenous retroviruses [44], significant cross-reactivity between the antibodies used and other viral proteins may be a factor here.

Taken together, we conclude that syncytin 1 and 2 and their cognate receptors play a substantial role in the cellular uptake of mitochondria, which seems highly specific and triggered mainly by syncytin1 and 2 HERV-envelope proteins than by their receptors.

## DISCUSSION

In this study, we demonstrate that cytotoxic stress by etoposide induces accumulation of mitochondria in human U87 glioblastoma cells, which were associated with different HERV-envelope proteins including, significantly, HERV-WE_1_ (syncytin-1) and HERV-FRD_1_ (syncytin 2). Moreover, mitochondrial preparations also contained the cognate receptors, i.e. ASCT2 for syncytin 1 and MFSD2 for syncytin 2. Unexpectedly, in these mitochondrial fractions some HERV-RNA transcripts were also detected. Upon co-cultivation of cells and free isolated mitochondria, we found that mitochondria and associated syncytins 1/2 are directly taken up by the cells across the cell membrane. In an effort to elucidate the role of syncytin 1 and syncytin 2 in this phenomenon, we were able to block this mechanism using specific antibodies against these HERV-envelope proteins. We therefore conclude that syncytin 1 and 2 as well as their cognate receptors are critical to the cellular uptake of mitochondria.

To our knowledge, neither the association of HERV-envelope proteins and mitochondria nor the mitochondria-mediated transfer of HERV proteins has been previously described. Moreover, we detected HERV co-localization to mitochondria also in other cancer cell lines including colon carcinoma, ovarian carcinoma, testicular cancer, etc. (unpublished). Our data suggest that the intercellular exchange of mitochondria via cell membranes depends on HERV-envelope proteins like syncytin1 and syncytin 2, since specific blocking inhibits this mechanism.

### Syncytin and cell fusion

Cell-cell fusion events are controlled by different proteins expressed by the fusing cells [46, 47]. Cell fusion proteins (fusogens) have been investigated for decades. Syncytins represent a family of diverse proteins that originated from HERVs related to the HIV gp41 envelope glycoprotein. Both endoretroviral syncytins, syncytin 1 and syncytin 2, are sufficient to induce cell-cell fusion in different cell lines in a receptor-dependent manner via disulfide bridge-forming CX_2_C and CX_7_C motifs, that are essential to their fusogenic activities [9]. Syncytin 1-mediated membrane fusion requires an interaction between this HERV-envelope protein and its receptor in the target membrane.

Excellent reviews on cell fusion, syncytins, and possible implications for cancer therapy were recently published [46–48]. Although not yet reported for mitochondrial fusion, it is reasonable to hypothesize that syncytin 1 may interact with the type-D mammalian retrovirus receptor SCL1A5 as their functional receptor described for placental cell fusion. In addition, MFSD2 was identified as the receptor for syncytin 2 [48, 49].

### Mitochondria and cellular cross-talk

Recent publications highlight that extracellular vesicles (EVs) may represent an important micro-environmental factor possibly modulating the tumor biology and chemoresistance [50]. The release and uptake of EVs by a number of different cell types leads to the transfer of various biomolecules including proteins, lipids, and nucleic acids [51]. A body of studies shows that EV cargo content such as mRNA may promote biochemical changes in the recipient cells [52–54]. Mitochondrial transfer has already been described in various models [29, 31, 55]. Furthermore, the preferential transfer of mitochondria from endothelial to cancer cells through tunneling nanotubes was recently reported to modulate chemoresistance [29]. In addition, high mitochondrial mass was identified in a subpopulation of stem-like, chemoresistant cancer cells [34]. Based on our data, it is conceivable that some cancer cells may also enhance chemoresistance by increased expression of HERV-envelope proteins. Both syncytin 1 and 2 seem to have a high affinity to mitochondria. Although the localization of these HERV-proteins with regard to mitochondrial membranes is still unclear, we speculate that syncytin 1 and 2 are localized in the outer membrane, because blocking antibodies prevent cellular uptake via cell membrane.

### HERVs and cancer

Although most HERV proviruses have accumulated replication-inactivating mutations or epigenetic modifications, many do still contain intact open reading frames (ORFs) encoding retroviral proteins [2, 10] that contribute to cellular transformation [12, 56]. Indeed, it has become evident that some HERVs possess properties that might be conducive to, or facilitate cancer development and biology, because we often find HERV expression to be elevated in transformed cells. In fact, tumor-restricted expression has been proposed for several HERVs [20]. This HERV expression is subject to both cell-intrinsic and extrinsic factors [5, 7]. While the relationship between human cancer biology and HERV activity is not fully understood. However, products of the envelope genes, i.e. syncytins, which are normally restricted to the placenta, have been identified in human malignancies, and it has been suggested that syncytin-mediated cell fusion events play a role in the transformation and metastasis process [21, 24, 33, 46, 57].

For instance, HERV-K expression has been correlated with the prognosis and progress of hepatocellular carcinoma [23], and is strictly required to sustain CD133^+^ melanoma cells with stemness features [17]. Recently, it was reported that activation of HERV-K envelope protein is essential to tumorigenesis and metastasis formation in breast cancer [24]. Furthermore, our own group has linked enhanced HERV expression is to the development of chemoresistance in colon carcinoma cells [25].

Both fusogenic glycoproteins, syncytin 1 and syncytin 2 are HERV-derived entities that are mainly expressed in human placenta under physiological conditions [58, 59]. In pathological settings, syncytin-1 has been reported to be expressed in testicular, brain, and breast tumors [11, 14, 20, 48, 60]. Moreover, downregulation of syncytin 1 expression inhibited carcinoma cell fusion [61]. Cancer-cancer and cancer-host cell fusion events have been linked to syncytin expression [22, 62]. Syncytin 1 has also been reported to exert an anti-apoptotic influence in staurosporine-stressed cells, possibly by decreasing activation of caspase 3 and increasing the expression of Bcl-2 [63]. This observation is consistent with our results, as we detected enhanced expression of the HERV-WE_1_-derived protein syncytin-1 as well as the HERV-FRD_1_-derived protein syncytin-2 upon induction of cytotoxic stress with etoposide, if only in mitochondrial preparations.

### Syncytins and cellular uptake of mitochondria upon induction of cytotoxic stress with etoposide

Apart from cancer-cancer cell fusions, altered mitochondrial dynamics, i.e. altered rates of mitochondrial fission and fusion are a common feature in cancer [22, 29, 64–66]. These alterations can influence metabolic function, proliferation and cell survival [67, 68]. Mitochondrial fusion and fission dynamics are essential to various mitochondrial functions and are also involved in mitochondria-mediated apoptosis. Mitochondrial dynamics are controlled by a number of intracellular proteins, including fusion (Opa1 and mitofusins 1 and 2) and fission proteins (Drp1 and Fis1), which may be pro-apoptotic or anti-apoptotic depending on cell type, status, and micro-environmental stimuli. For instance, dysregulated mitochondria via defective p53 or Oma1 may to some degree be involved in the pathophysiology of cisplatin resistance [30, 45, 64, 69, 70]. HERV proteins have not been reported to be implied in mitochondrial dynamics to date. We previously reported on chemo-resistant, highly aggressive cell populations that presented cancer stem cell markers, aberrant signaling pathways, and a set of deregulated miRNAs [32]. Furthermore, resistant tumor cells displayed mitochondrial accumulation around the nucleus in response to cytotoxic stress induced by etoposide. This phenomenon was correlated with intensified intracellular and intercellular mitochondrial traffic as well as the uptake of free extracellular mitochondria. Our results support recent reports of augmented mitochondrial mass in chemoresistant, stem-like breast cancer cells [34]. The authors suggested a new mitochondria-based model for understanding the relationship between cancer stem cells and chemoresistance in which mitochondria confer protection against, or increased repair of, DNA damage.

The observed mitochondrial dynamics might also regulate chemoresistance via “metabolic reprogramming”. Mechanisms of mitochondria-mediated cancer stem cell resistance to chemo- and radiotherapy and mitochondrial function in the context of cancer drug resistance has recently been discussed in numerous reports [34, 68]. Metabolic reprogramming has been recognized as a hallmark of cancer, as tumor cells depend on the mitochondrial metabolism as well as aerobic glycolysis. Mitochondrial transfer is a critical mechanism for tumor cells with defective mitochondria to restore oxidative phosphorylation. This process is quintessential for promoting tumor progression especially in cancer cells without intact mitochondrial DNA [69].

Nevertheless, we can now correlate the cytotoxic stress-triggered cellular uptake of mitochondria to certain HERV proteins, specifically syncytin 1 and syncytin 2, together with their cognate receptors, i.e. SLC1A5 and MFSD2 that we detected in mitochondria.

We conclude that the association of mitochondria with both syncytin-1 and syncytin-2, together with the newly identified cell-to-cell transfer of mitochondria-associated HERV-specific proteins may contribute to the rise of resistance against anti-cancer therapy. Bypassing the mechanisms of mitochondria-mediated resistance appears to be an attractive avenue to new mitochondria-targeted therapies, with molecular candidates including different antiviral drugs that are already available [25].

## MATERIALS AND METHODS

### Cell cultures

Cells employed in this study were obtained from the cell and tumor bank of the University of Duisburg Essen, Medical School. Wildtype (WT) and etoposide-resistant cells were cultured in DMEM medium (Invitrogen, Karlsruhe, Germany) containing 10 % heat-inactivated fetal calf serum (FCS) and 15 μg/ml Ciprobay (Bayer AG Wuppertal, Germany).

### Induction of mitochondrial accumulation in the perinuclear space using etoposide

Near-confluent U87 human glioblastoma cells were stressed with 5 μg/ml etoposide (2x IC_50_) for at least 10 days. After this, a dense mass of mitochondria in the perinuclear space could be directly observed by light microscopy. Cells could be cultured in these conditions for long periods if the medium was replaced from time to time. With slight differences, metaplastic phases and cell hypertrophy were maintained in cell culture for long periods. Mitochondrial accumulation was visualized by incubating the cells with MTT to a final concentration of 1 mg/ml for 30 minutes prior to documentation.

### Induction of etoposide-resistant cells

Resistance induction was performed by the selection of subpopulations using etoposide as previously described [32]. Briefly, IC_50_ values for etoposide were determined by MTT assay. Exponentially growing cells were then exposed to 2x IC_50_ etoposide for 24 h. For recovery, the cells were washed and incubated in drug-free culture medium until new colonies had formed. This procedure was repeated several times, each time doubling the original IC_50_ until 64x IC_50_ was reached. The surviving cells were subjected to resistance selection by incubation with increasing concentrations of the respective drugs (2x to 16x IC_50_) for 24 h. Cells that proliferated at higher drug concentrations (8x) within 1 week were considered chemotherapy refractory. Resistant colonies were then expanded in the continuous presence of etoposide and used for molecular-biological analysis. The resistance factor (RF) was determined by MTT proliferation assay and reported as the IC_50_ resistant/IC_50_ parental ratio.

### Isolation of mitochondrial and nuclear fractions

Mitochondria and nuclei were isolated according to standard protocols permitting the recovery of functionally intact organelles [45]. U87 mitochondria-donor cells were grown to 90 % confluence. The medium was discarded, and cultures were rinsed twice in cold PBS. Then, PBS was completely removed and 1.4 ml pre-chilled lysis buffer (0.01 mol/l Tris-HCl pH 7.8, 0.001 mol/l EGTA, 0.250 mol/l sucrose, 1 % BSA) was added while keeping the flasks on ice for a few minutes. Cells were detached with a scraper, transferred to a pre-chilled Dounce homogenizer and incubated on ice for another 15 minutes. Afterwards, they were homogenized on ice applying 40 strokes. Cell homogenates were transferred to 2 ml Eppendorf tubes and centrifuged for 5 minutes at 2500 g, 4°C. The upper phase was transferred to 2ml tubes; the pellets (nuclear fraction) were resuspended in 700 μl lysis buffer, transferred to the Douncer, homogenized, and spun down in the same conditions. The upper phase containing the mitochondrial fraction was centrifuged for 5 minutes at 5000 g, 4°C and the resulting pellet maintained on ice. Supernatants were transferred to new 2ml tubes and examined by light microscopy for nuclear contamination. The mitochondrial fraction was spun down for 15 minutes at 10000 g, 4°C and the mitochondrial pellet washed once in 700 μl lysis buffer and centrifuged at 10000 g for 15 minutes. A small portion of the mitochondrial and nuclear fractions were lysated in RIPA buffer, and the protein content was determined using the Pierce BCA Protein Assay Kit (Thermo Scientific Inc, MA, USA). In order to measure the organelle content, the protein concentration was adjusted to 10 mg/ml. Physiological experiments were performed immediately after organelle isolation. Protein extracts were portioned and stored at -80°C for WB analysis. For cell experiments, mitochondrial fractions were resuspended in 1.6 ml DMEM medium, added to the cultures and incubated at 37°C.

### RNA purification and cDNA synthesis

Total RNA was extracted with Trizol^®^ (Life Technologies, California, USA) based on standard protocols [25]. To eliminate genomic DNA contamination, the eluted RNA containing 10 IU RNase inhibitor was treated with 7 Kunitz units of RNase-free DNase I (Qiagen, Hilden, Germany) in an appropriate buffer and incubated at 25°C for 20 minutes. RNA samples were then further purified on RNeasy mini columns (Qiagen, Hilden, Germany) according to the manufacturer's instructions. RNA integrity was ascertained by agarose gel electrophoresis and densitometric analysis. 1 μg of pure and intact RNA was used for first-strand cDNA synthesis using the cDNA Reverse Transcription Kit from Life Technologies, following the kit instructions.

### Real-time PCR and primers for HERV detection

The amplification of 25 ng of RNA was performed in triplicate in a CFX96™ Real-Time System (Biorad Laboratories, California, USA). Results were analyzed with the CFX-Manager™ Software Version 3.1 (Biorad Laboratories, California, USA).

All primers were designed and synthesized by RealTimePrimers.com (PA, USA). RNA sequences were obtained from the database of the US National Library of Medicine, National Institute of Health. HERV-V1, HERV-V_3-1_, HERV-WE_1_ (syncytin 1) and HERV-FDR_1_ (syncytin 2) expression was confirmed using validated TaqMan primers and probes from Life Technologies (CA, USA), using TaqMan PCR core reagents according to the manufacturer's recommendations. Primer details are summarized in Table 1.

**Table 1:** Real-time PCR primers used for the detection of HERVs

Symbol	Accession	Region	Forward	Reverse	Size _(bp)_
HERV-W_E1_	AF072506	290-463	gggttccatggttctcttct	tggtgaaccacttccaagat	174
HERV-3_1_	NM001007253	1377-1562	taaccagaaattgcctgagc	gaagaggcggttagtgtgaa	186
HERV-V_1_	NM152473	1565-1757	acacctctgaggagggattc	cccaggggaacagtagattt	241
HERV-H_1_	BC015108	543-778	caccccaacacttcaacact	tcacttaaggcaaggactgg	236
HERV-E_1_	BC037342	1398-1562	tacctactctggtgggtgga	ctttgccctcttctgtacca	165
HERV-K10	FN806835.1	241-421	ttgggctagtcaatgtcgtt	ctggcttattccctgaaaca	181
HERV-FRD_1_	NM207582	504-698	ctcattctcacgccttcact	taattccgcctctatgcttg	195
HERV-F (*pol*)	AB120695	98-229	tacagcagcagcagcagttt	atctgggaaggaggaggaga	132
HERV-S (*pol*)	AB162179	81-264	ccaacgtgttacctccactc	cagttcccgataatccactg	184
HERV-T (*env*)	AB266802	690-920	cataatttgccggtcatagg	agttgatcccccagagtagg	231
HERV-L	EF141078	132-245	agggctattatggtgggaag	catctttcaggtccttggtg	123

The accession, region, sequence, polarity and product size for the primers used are reflected.

### Mitochondrial labeling with MitoTracker^®^

To track exogenous mitochondria and their cellular uptake, organelles extracted from U87 cells were labeled with 25 nM green or red MitoTracker^®^ (Life Technologies, Darmstadt, Germany) in serum-free medium at 37°C for 120 minutes [32]. Cells were washed several times in order to remove free MitoTracker^®^.

### Mitochondrial labeling with anti-syncytin-1-FITC conjugated antibody

To track exogenous mitochondria and their cellular uptake, mitochondria extracted from U87 cells were blocked with 10 % BSA for one hour. The organelles were then centrifuged, labeled with anti-syncytin-1-FITC conjugated antibody Biorbyt Cat. No. orb111912 (Cambridge, UK), diluted to 1:10 in 1 % PBS-BSA, and incubated at room temperature for 120 minutes. Samples were then washed twice in 1 % PBS-BSA, re-suspended in DMEM containing 10 % FCS, and added to the cultures.

### Labelling of exogenous mitochondria with anti-syncytin 1 and anti-syncytin 2 antibodies

Free mitochondria were labelled with anti-syncytin 1 and-syncytin 2 antibodies (Bioss, USA, Cat. No. bs2962R and bs15466R) to block mitochondrial entry into U87 cells. The mitochondrial fraction was blocked using 10 % of BSA dissolved in PBS for 30 minutes. Anti-syncytin antibodies diluted 1:10 in 1 % BSA were applied for one hour at 37°C. Mitochondria were washed twice as described above. Syncytin-blocked mitochondria were then added to U87 cells. After 24 hours, the concurrence of exogenous and endogenous mitochondria was monitored using a Nikon Eclipse Ti fluorescence microscope.

### Western blot analysis

Mitochondrial pellets were lysed in RIPA buffer [150 mM NaCl, 1 mM EDTA, 1 % Triton X-100, 1 % sodium deoxycholate, 0.1 % SDS, 50 mM Tris-HCl pH 7.4] in the presence of a proteinase inhibitor cocktail according to the manufacturer's instructions (Roche Diagnostics GmbH, Mannheim, Germany) on ice for 30 minutes and then centrifuged for 20 minutes at 14000 g, 4°C. Supernatants (30 μg) were resolved by SDS-PAGE in a 4-12 % gradient gel (Invitrogen, Karlsruhe, Germany) using Tris-glycine SDS (0.025 M Tris-HCl, 0.192 M glycine, 0.1 % SDS w/v, pH 8.3) buffer, and transferred to 0.2 μm nitrocellulose membrane (Pierce Protein, Thermo Fisher Scientific Inc., MA, USA). Blots were blocked with 5 % BSA or non-fat milk taking into consideration the recommendations of the manufacturers of the primary and secondary antibodies. Immunoblots were developed by Western Lightning^®^ Plus-ECL (Perkin Elmer, CA, USA) using a ChemiDoc XRS+ system with Image Lab software version 2.0.1 (Biorad, CA, USA).

### Antibodies used in immunological techniques

Primary antibodies were purchased as follows: anti-HERV-WE_1_ (syncytin 1) and anti-HERV-FRD_1_ (syncytin 2) from Bioss, USA (Cat. No. bs2962R and bs15466R) and Biorbyt, UK (Cat. No. orb111912); anti-lamin A+C from Novus Biologicals, USA (Cat. No. EPR4100); Cox IV (Cat. No. 11967), ABCG2 (Cat. No. 4477), MFN1 (Cat. No. 13196), MFN2 (Cat. No.9482), Bcl-2 (Cat. No. 2870), Bcl-X_L_ (Cat. No.2764), BAD (Cat. No.9239) and BAX (Cat. No.5023) primary antibodies from Cell Signaling Technology, (USA); polyclonal antibodies targeting SLC1A5 (Cat. No. bs-0473R) and MFSD2A (Cat. No. bs-6073R) from Bioss, USA; conjugated secondary antibodies from Cell Signaling (USA) and Jackson ImmunoResearch Europe Ltd. (Suffolk, UK).

### Detection of mitochondrial populations by fluorescence flow cytometry

Fluorescence flow cytometry was performed to determine the relative percentages of HERV elements in the mitochondrial fraction. Samples were labeled overnight at 4°C against both Cox IV and HERVs. Secondary antibodies conjugated with FITC and APC (Thermo Fischer Scientific, Pittsburgh, USA) were used for HERV and CoxIV detection, respectively. For each experiment, cells were stained with appropriate isotype control antibodies to establish the level of background staining and set quadrants before calculating the percentage of positive HERV classes present in the mitochondria. Detection was performed using the CytoFLEX Research Cytometer B5-R0-V0 (Beckman Coulter Biosciences, Krefeld, Germany) equipped with three different lasers tuned to 488 nm (50 mW) blue, 638 nm (50 mW) red, 405 nm (80 mW) violet, and filter set to detect different emission wavelengths. The FlowJo 10.0 Analysis Software (Tree Star, Oregon, USA) was used to quantify the positivity of samples.

### Immunocytochemical (ICC) staining

ICC staining was performed according to standard protocols with some modifications [25, 32]. Briefly, ICC cells were grown on chamber slides to appropriate densities, washed with 1x PBS, fixed with 4 % formaldehyde in PBS for 20 minutes, rinsed twice with 1x PBS for 5 minutes, and blocked with 10 % normal goat serum (AbD Serotec, London, UK) at room temperature for 60 minutes. All primary antibodies were applied according to the manufacturer's recommendations. Sections were incubated overnight in PBS/0.05 % Tween 20, 1.5 % goat serum at final concentrations between 1 and 5 μg/ml. On the next day, the slides were washed 3x with PBST (PBS/0.05 % Tween 20) for 5 minutes. Conjugated secondary antibodies diluted in PBS/0.05 % Tween 20/2.5 % goat serum were incubated for 120 minutes at room temperature according to the manufacturer's recommendations. Next, the samples were stained for 15 minutes with 10 μg/ml propidium iodide or Hoechst 33258 diluted in PBST (PBS/0.05 % Tween 20) in order to visualize the nuclei. Slides were then washed 3x in PBST for 5 minutes each. Tissue specimens were mounted in Faramount Mounting medium (Dako) for visualization.

## SUPPLEMENTARY MATERIALS FIGURES





## Abbreviations

HERV: human endogenous retrovirus
DMEM: Dulbecco's Modified Eagle Medium
WT: wildtype (referred to cells)
RETO: resistant to etoposide (referred to cells)
RF: resistance factor (referred to cells)
MTT: 3-(4,5-dimethylthiazol-2-yl)-2,5-diphenyl-tetrazolium bromide
ICC: immunocytochemical
PBS: phosphate buffered saline
BSA: Bovine serum albumin
FITC: fluorescein isothiocyanate
APC: allophycocyanin
RIPA: radio-immuno-precipitation Assay
SDS-PAGE: sodium dodecyl sulfate polyacry-lamide gel electrophoresis
EDTA: ethylenediaminetetraacetic acid
EGTA: ethylene glycol tetraacetic acid
PCR: polymerase chain reaction
FACS: fluorescence-activated cell sorting
EVs: extracellular vesicles
ABCG2: ATP-binding cassette sub-family G member 2
