# Supplementary material for: Cytotoxic stress induces transfer of mitochondria-associated human endogenous retroviral RNA and proteins between cancer cells
Source: Oncotarget. 2017 Oct 7;8(56):95945–64. doi: 10.18632/oncotarget.21606 (PMC5707072; doi:10.18632/oncotarget.21606)
Supplement: Supplementary file 1 [file oncotarget-08-95945-s001.pdf]

Cytotoxic stress induces transfer of mitochondria-associated human endogenous retroviral rna and proteins between cancer cells

SUPPLEMENTARY MATERIALS

Supplementary Biochemie 1:

See Supplementary File 1
