# Supplementary material for: Cytotoxic stress induces transfer of mitochondria-associated human endogenous retroviral RNA and proteins between cancer cells
Source: Oncotarget. 2017 Oct 7;8(56):95945–64. doi: 10.18632/oncotarget.21606 (PMC5707072; doi:10.18632/oncotarget.21606)
Supplement: Supplementary file 2 [file oncotarget-08-95945-s002.docx]

**Biochemistry 1**: differential expression of syncytins in U87 cells untreated (**UT**) and treated (**T**) with etoposide. Upon etoposide incubation large amounts of syncytins are found in the mitochondrial fraction.

U87^UT^ U87^T^ U87^UT^ U87^T^

**Mitochondrial fraction isolated from U87 cells**


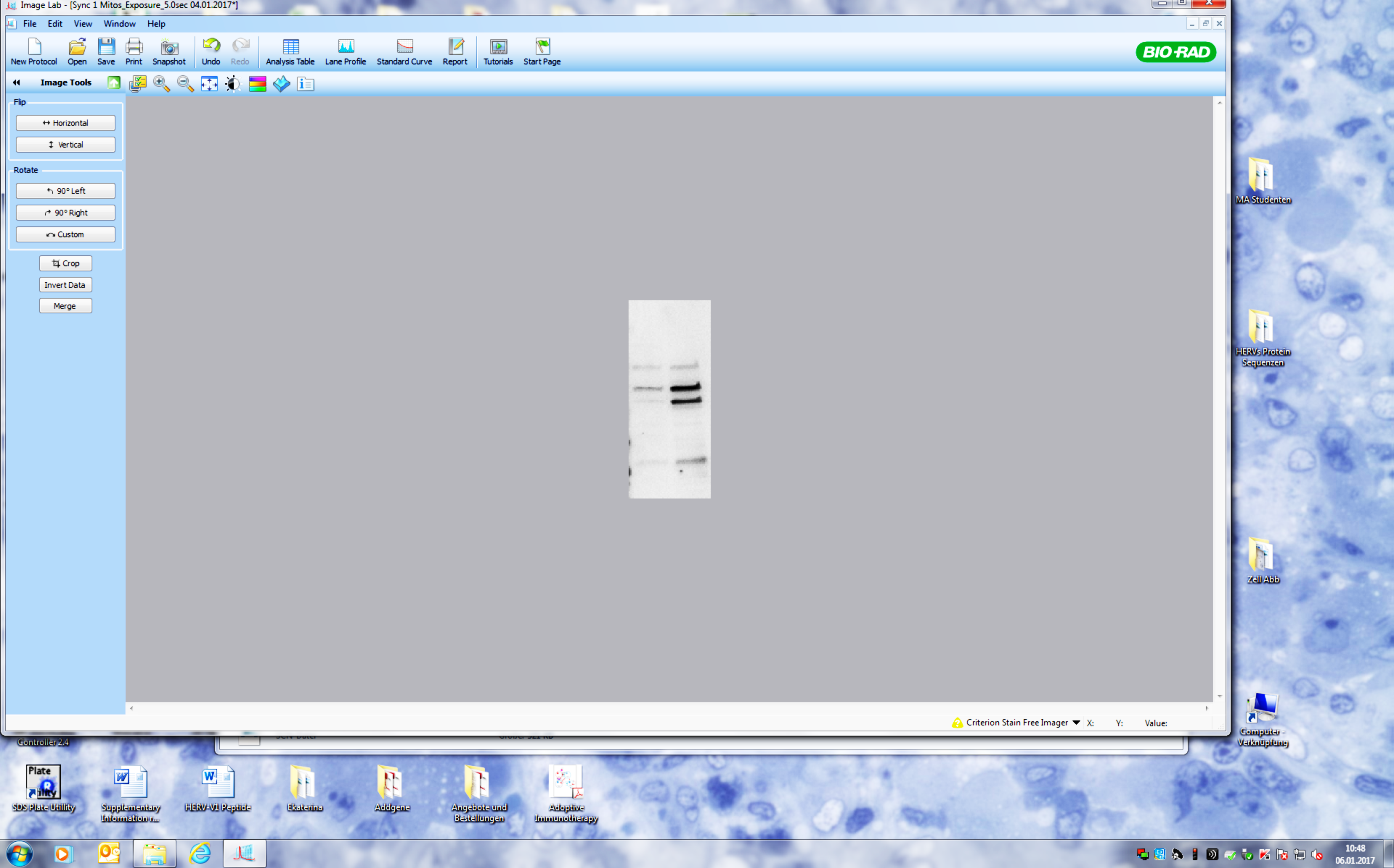

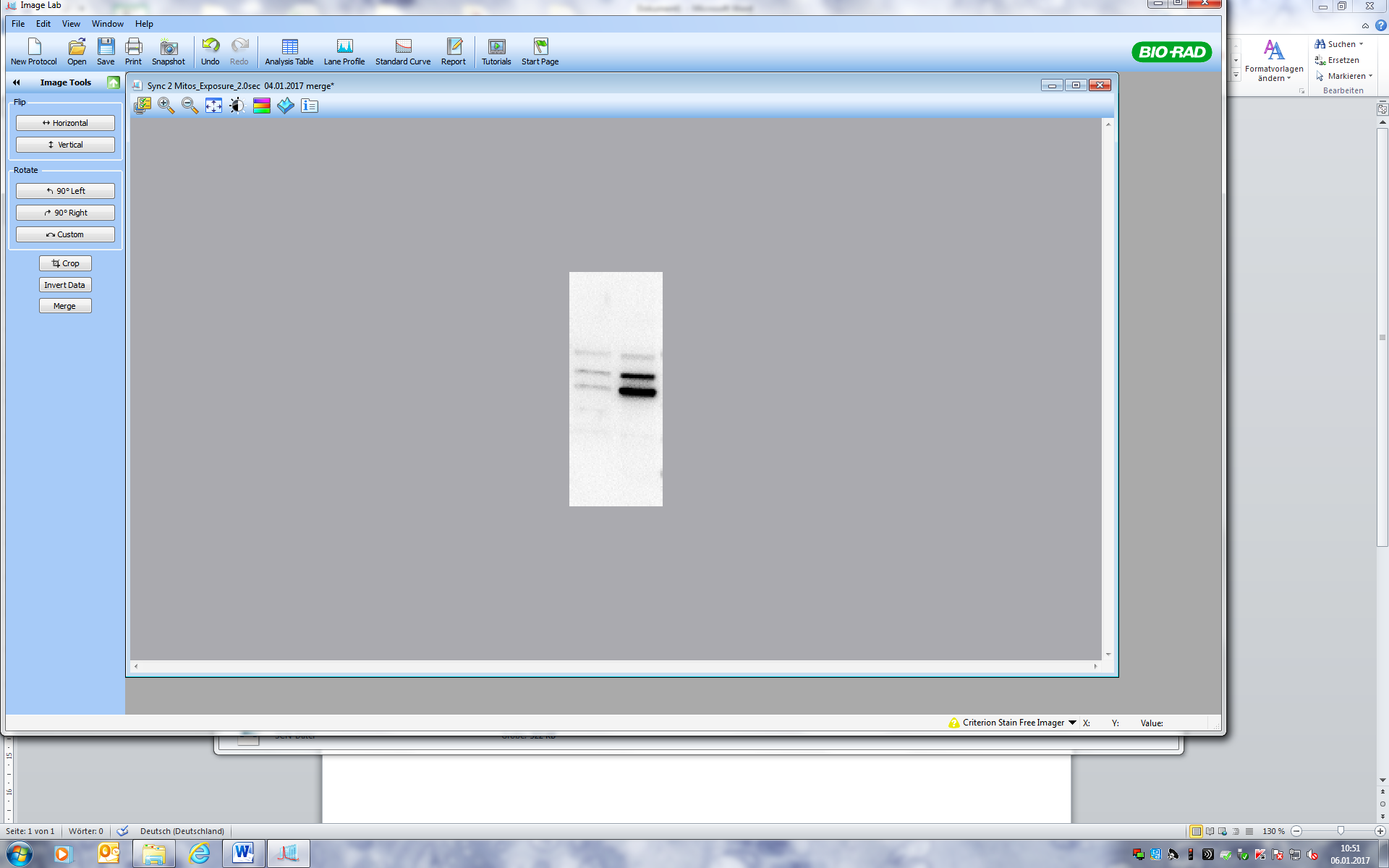


**HERV-WE_1_ HERV-FRD_1_**

135

75


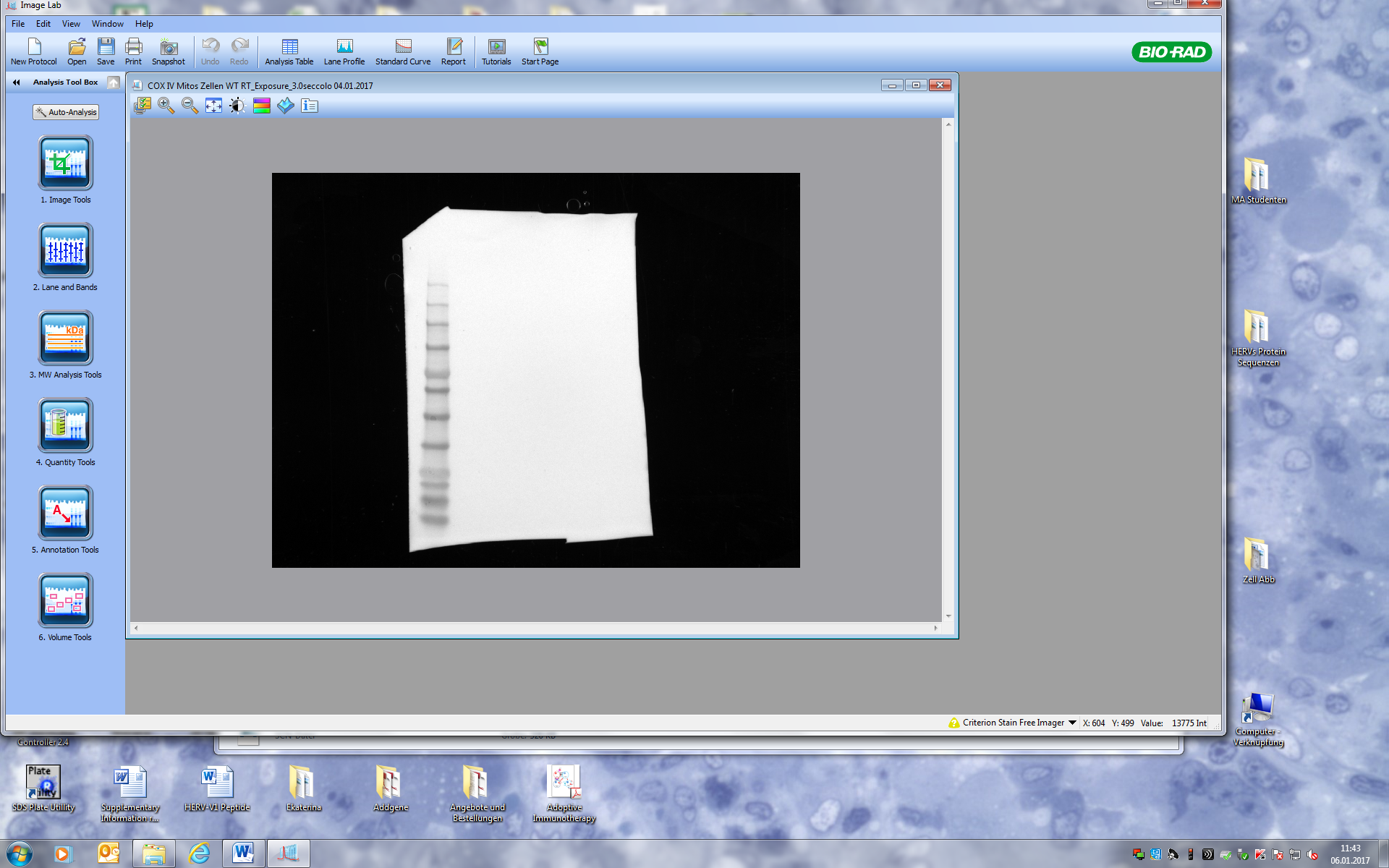


11

25

48

**Biochemistry 2**: No differences in the expression of Bax in the cytoplasm and whole cell extracts are detected. Cox IV was used as loading control.


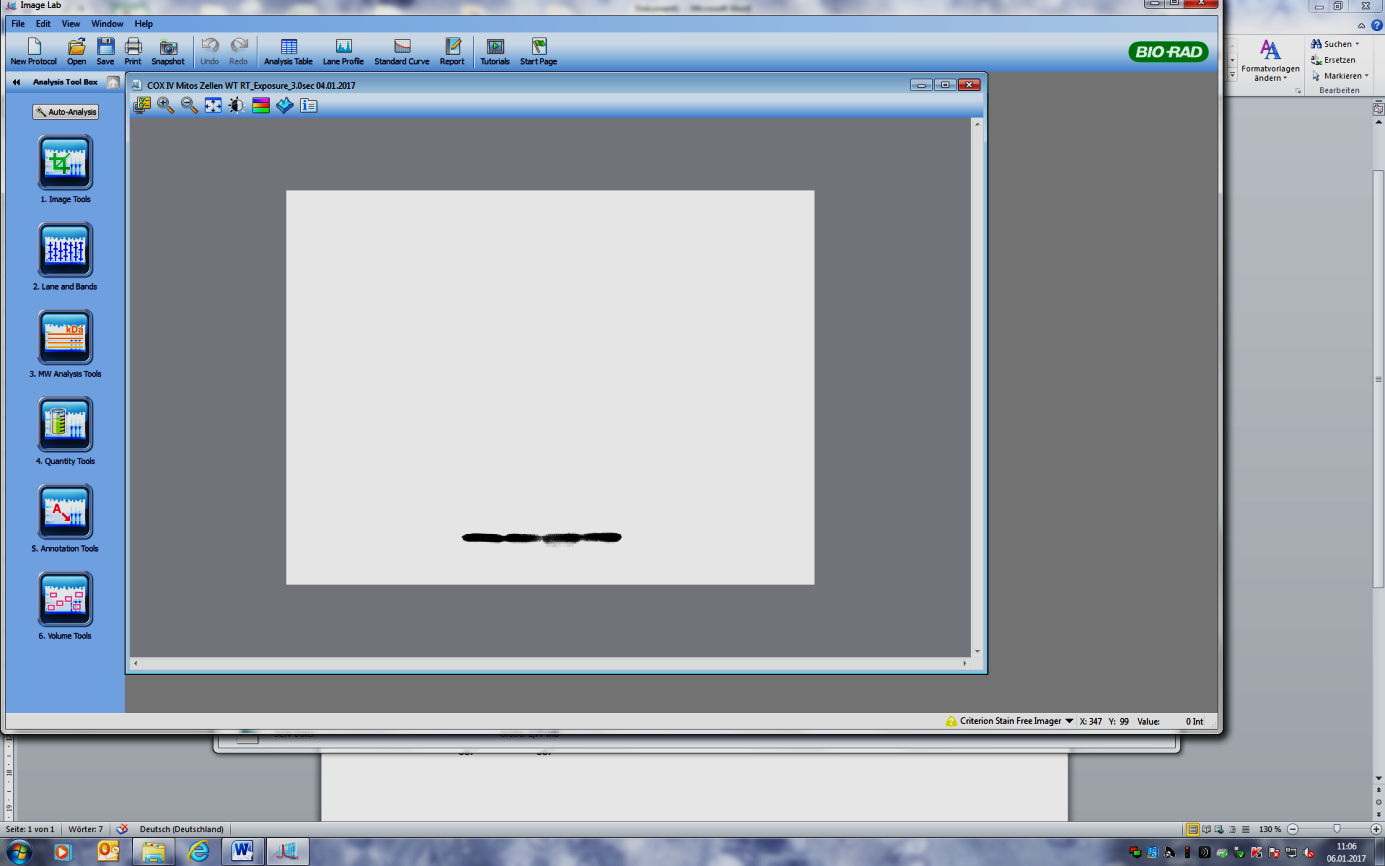


Mito Cell Mito Cell

U87^WT^ U87^RT^

Cell Cyt Cell Cyt

U87^WT^ U87^RT^


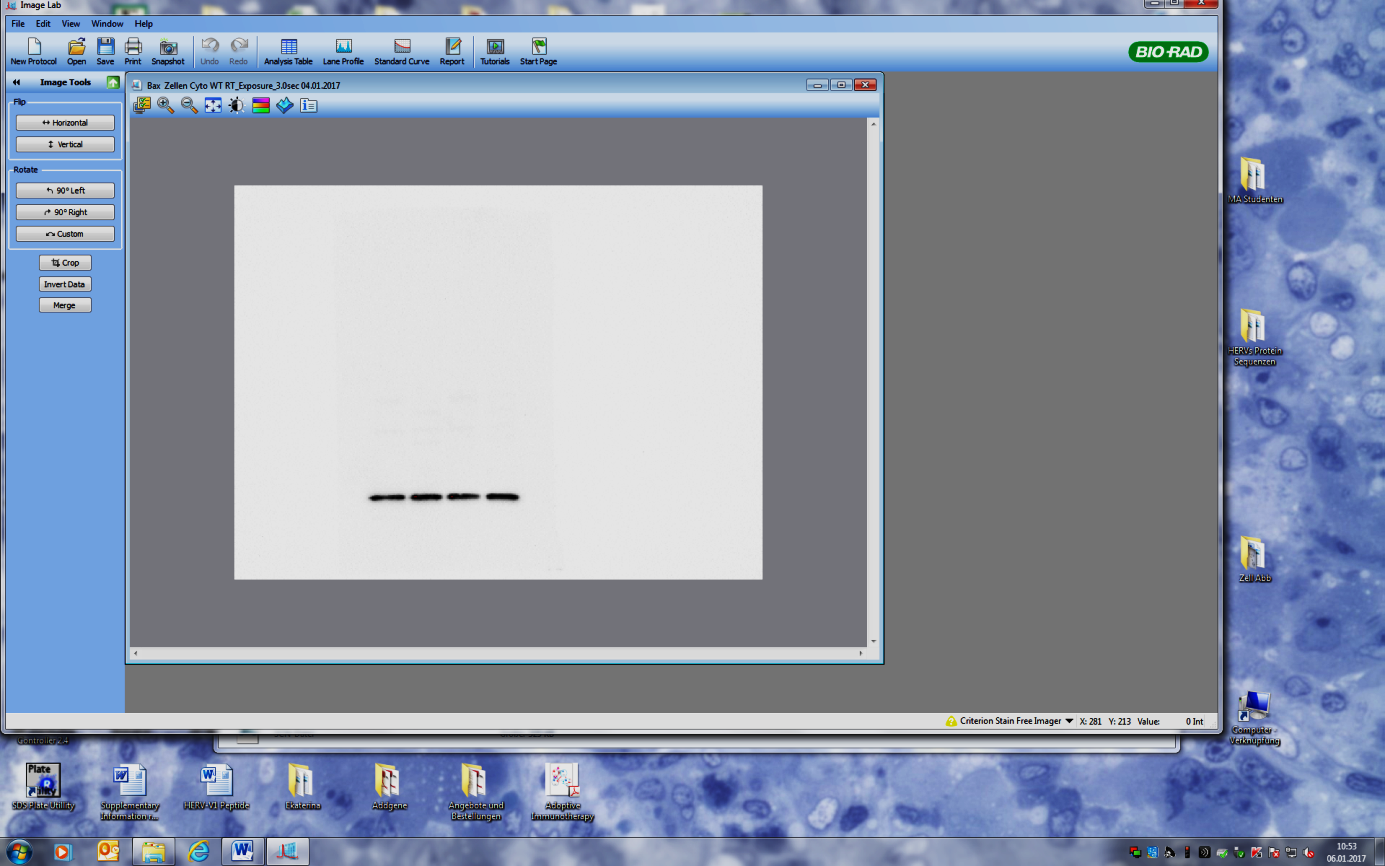


**Bax Cox IV**


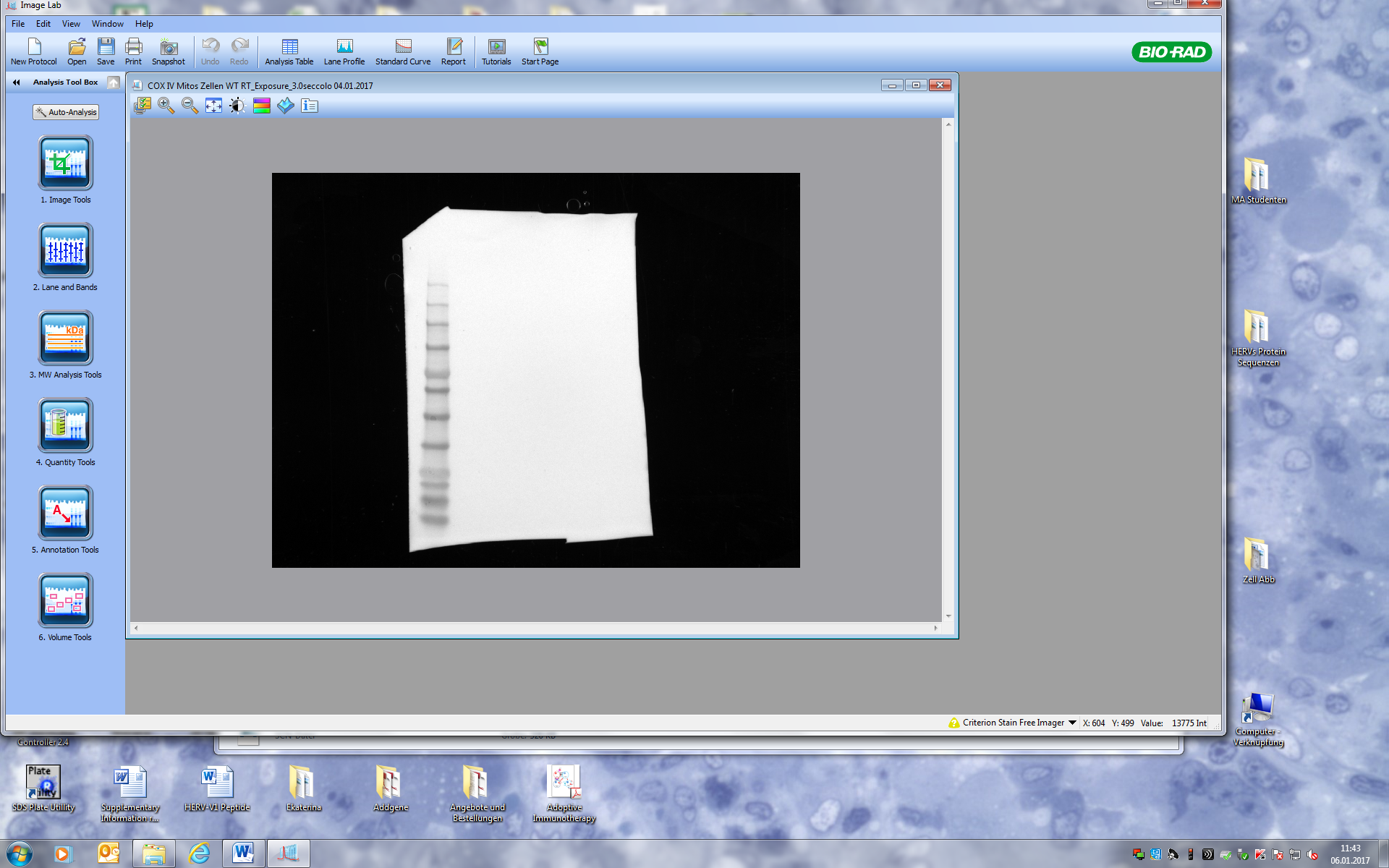


11

25

48

75

100

**Biochemistry 3**: Loading control of the sub-cellular fractions visualized by Coomasie protein staining.


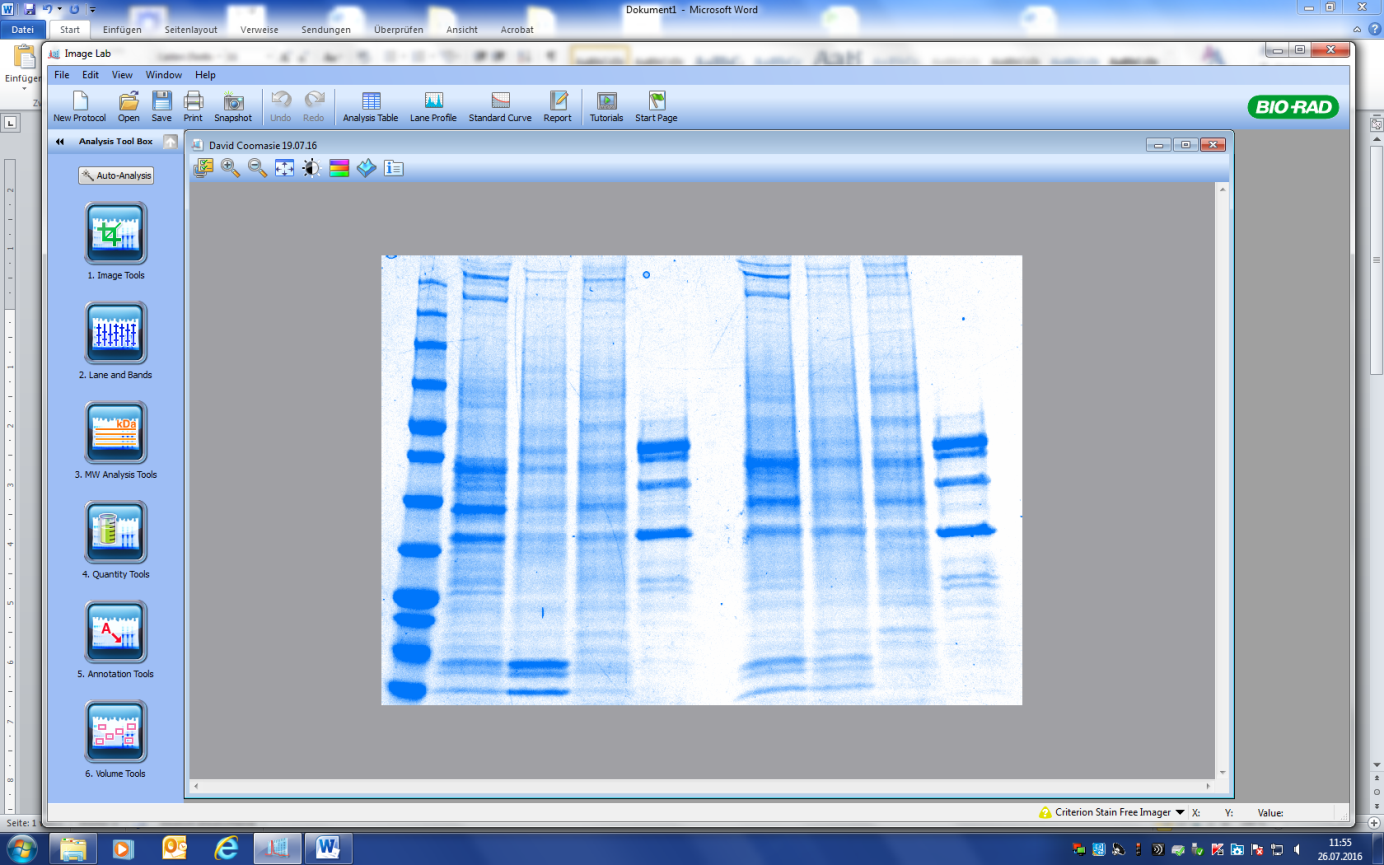


St Cells Nucl Mito Cyto Cells Nucl Mito Cyto

U87^WT^ U87^RETO^

17

25

35

48

63

75

100

135

180

**Biochemistry 4**: Detection of syncytins in the subcellular fractions of U87 cells. Coomasie staining for loading control.


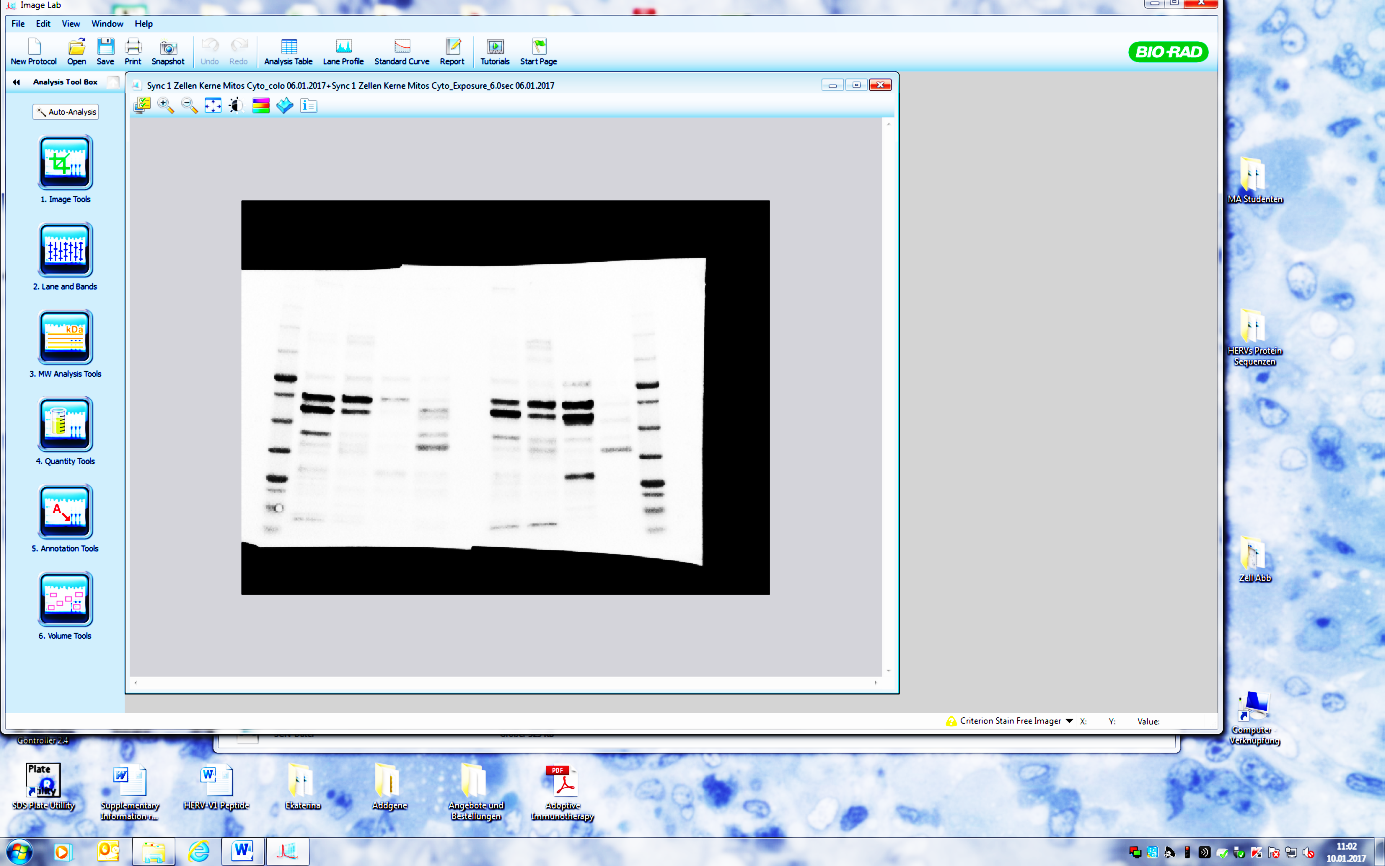


HERV-WE_1_ U87^UT^ HERV-WE_1_ U87^T^

St Cells Nucl Mito Cyto Cells Nucl Mito Cyto

11

25

48

75

HERV-WE_1_

53 KDa


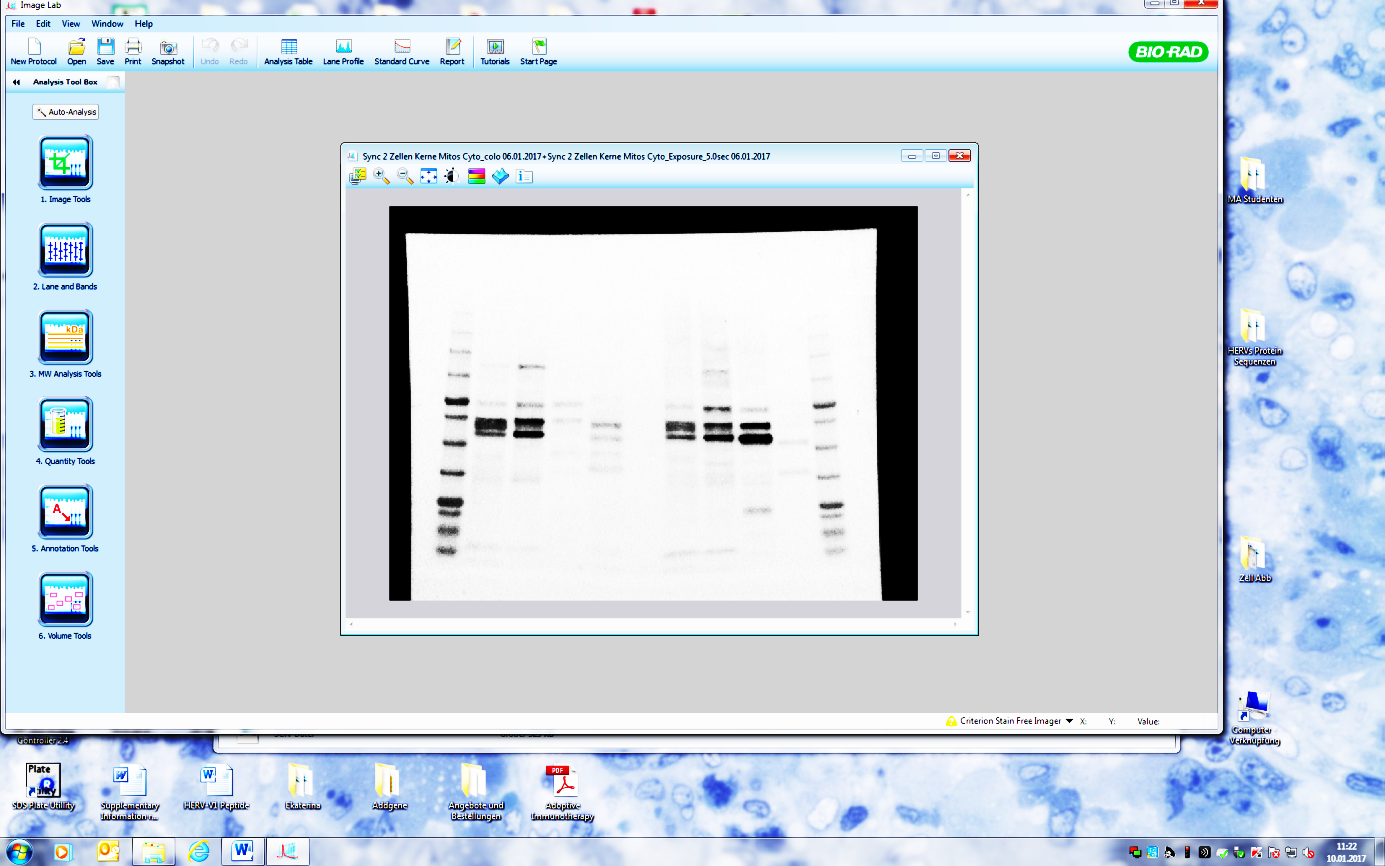


HERV-FRD_1_ U87^UT^ HERV-FRD_1_ U87^T^

St Cells Nucl Mito Cyto Cells Nucl Mito Cyto

11

25

48

75

HERV-FRD_1_

53 KDa

17

25

35

48

63

75

100

135


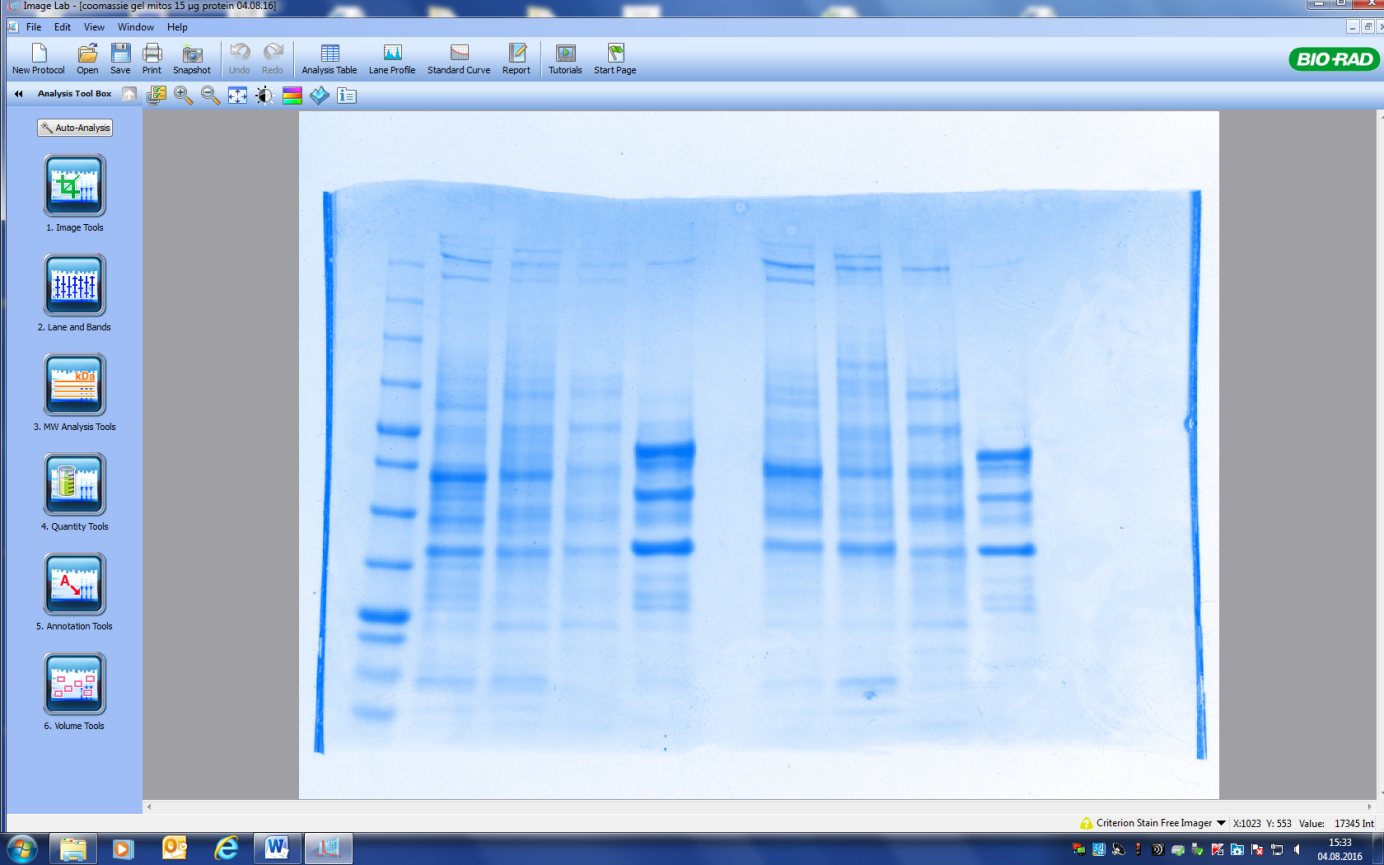

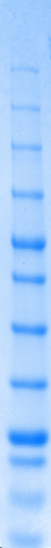


180

St Cells Nuclei **Mito** Cytop

**Biochemistry 5**: Expression of MFSD2 in U87 cells untreated (**UT**) and treated (**T**) with etoposide.

11

25

63

75


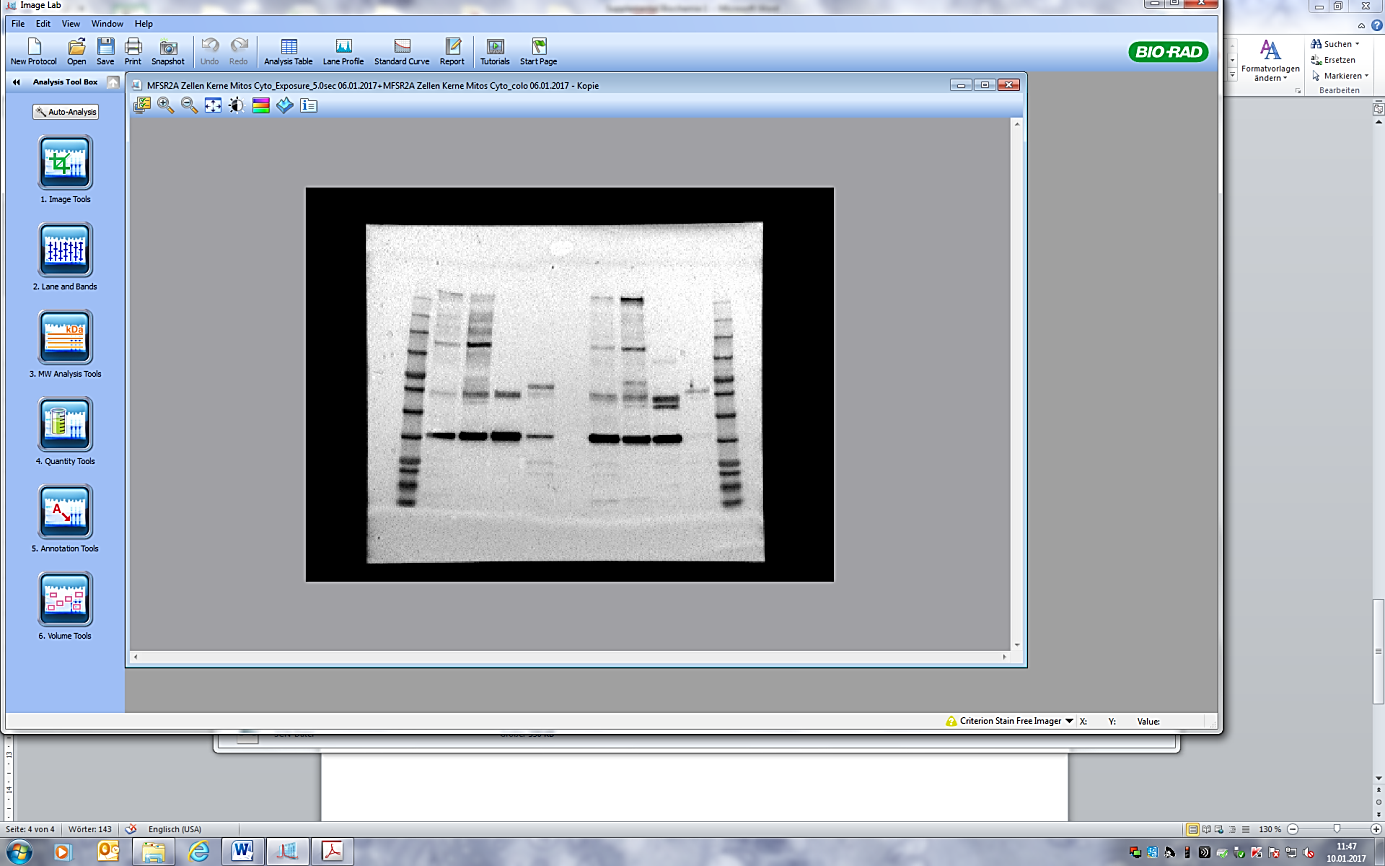


MFSD2 U87^UT^ MFSD2 U87^T^

St Cells Nucl Mito Cyto Cells Nucl Mito Cyto

MFSD2

55 KDa

MFSD2

36 KDa

**Biochemistry 6**: Measurement of cell hypertrophy in suspension. Etoposide-treated cells are very heterogeneous in respect to their size. Cells could reach up to 35 µm measured in suspension. Contrarily, untreated cells are very homogeneous in their size with a maximal size of 23 µm as measured in suspension form. This effect mirrors the planar hypertrophy shown by light microscopy. Increasing in cell death cells (red) is not observed after etoposide treatment at the doses revealed.


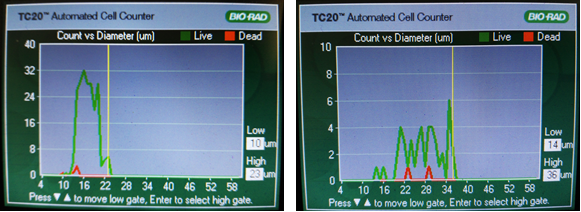


U87^UT^

U87^T^


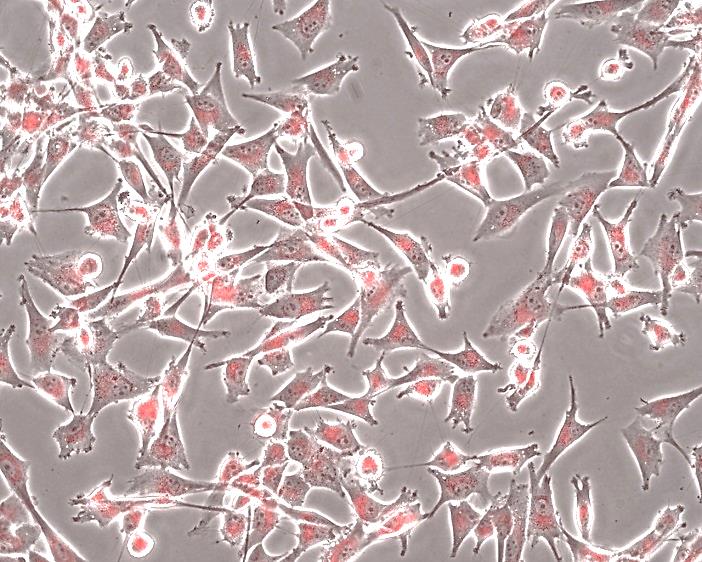


U87^UT^


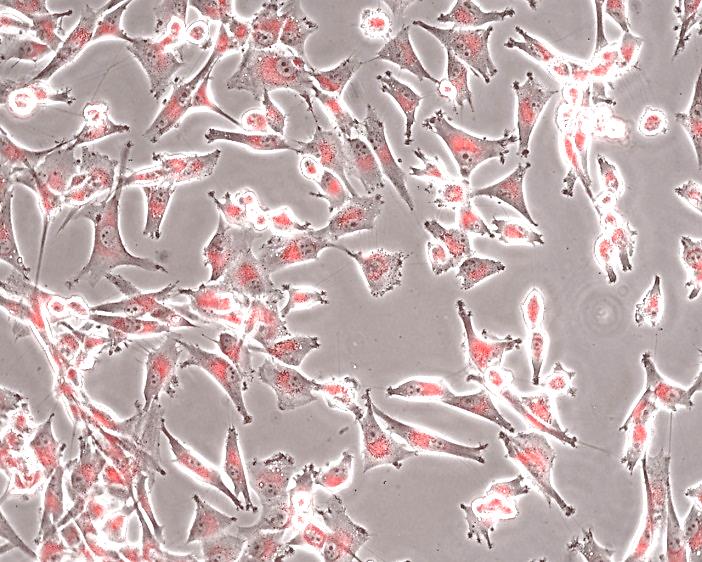


U87^UT^


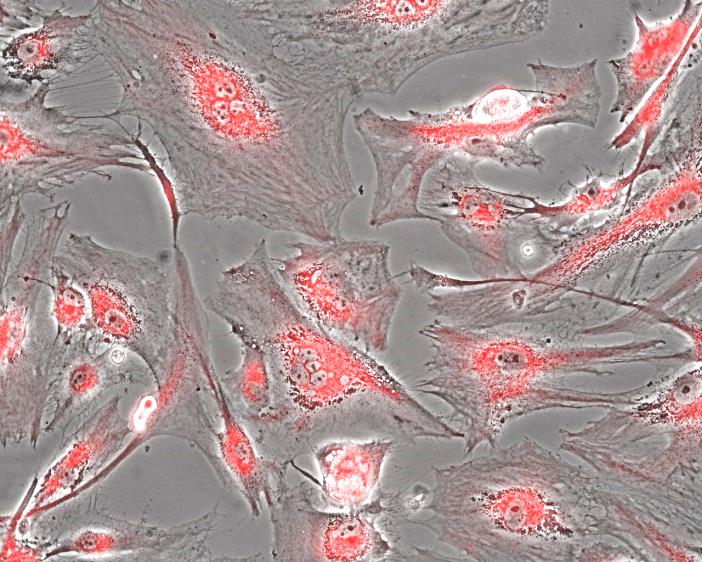


U87^T^


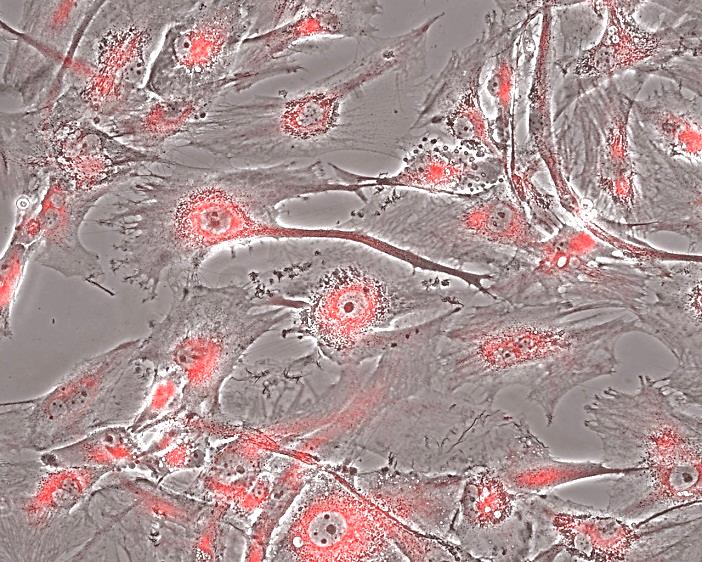


U87^T^

**Purity of mitochondria using sucrose gradients only or the combination of sucrose gradient and anti-TOM_22_ MicroBeads**: MB: anti-TOM_22_ antibody is covalently conjugated to magnetic beads.

Regarding the concern of possible contaminations of the mitochondrial fraction with cell membranes using only sucrose gradients, we undertook a double purification of mitochondria. For this purpose, we firstly isolated mitochondria using sucrose gradients as described in the Material and Methods section and subjected the mitochondrial fraction to a second re-purification of mitochondria using the Isolation Kit from Miltenyi Biotec, which based on an anti-TOM_22_ MicroBeads. (Cat. Nr. 130-094-532, Miltenyi Biotec, Bergisch Gladbach, Germany). This kit allows highly purified mitochondrial populations retaining its physiological fitness.

We did not observe any significant difference between the mitochondria isolated by sucrose gradient only and a consecutive re-purification of this fraction with the above mentioned kit.

Anti-TOMM_22_ Ab

N

S

Magnetic beads

TOMM_22_

Mitochondria

Mitochondrial fraction


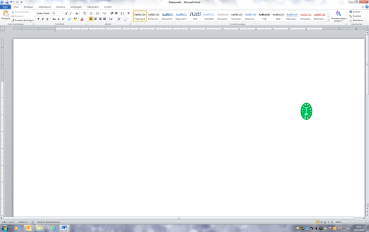

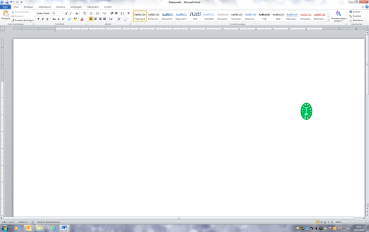

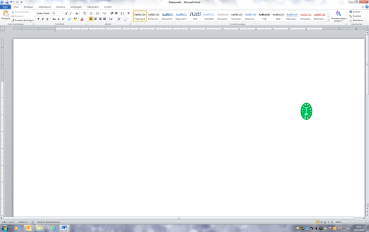

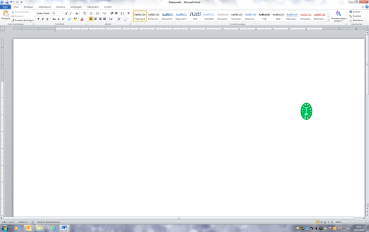


Mitochondrial pellets

Grandient separation Several whashings TOM_22_-magnetic-beads separation

**Supplementary Figure**: **Isolation of functional mitochondria from cells using magnetic beads**. Using the *Mitochondria Isolation Kit* (Cat. Nr. 130-094-532, Miltenyi Biotec, Bergisch Gladbach, Germany), cells are lysed and mitochondria are magnetically labeled with anti-TOM_22_ MicroBeads at 4°C for one hour. This antibody specifically binds the human TOM_22_. The cell lysate is loaded onto a MACS Columns, placed at a MACS Separator. The magnetically labelled mitochondria are retained within the colum, whereas the unlabelled organelles and cell components run through. Columns are then 3 times washed and the magnet retained mitochondria are eluted and recovered by centrifugation at 13 000 x g for 2 minutes. For proper analysis of the eluates, mitochondrial pellet was subjected to Westernblot for the detection of specific mitochondrial antigens like CoxIV.

We performed additional experiments checking for two different plasma membrane markers in the revised manuscript (see supplemental data in the revised manuscript as well as figures below). The first protein is ABCG2 that is exclusively localized in the cytoplasmic membrane (the protein is an ABC-transporter, essential for the traffic of organic and inorganic molecules across the cellular membranes). The second protein analyzed is Programmed death-ligand (PD-L1), also exclusively localized in the cytoplasmic membrane. PD-L1 is a 40kDa type 1 transmembrane protein, which has been shown to be an immuno-checkpoint regulatory protein, and being extensively expressed in glioblastoma cells. We did not select tubulin for quality check, because mitochondria are dynamic organelles and move inside the cell along microtubules (which are polymers of tubulin) and actin tracks. Therefore, in the process of mitochondria isolation, these organelles may contain cleaved tubulin or actin proteins.

Resuming, we could clearly show that both, ABCG2 protein as well as PD-L1 are only detectable in the whole cell fraction and in the plasma membrane fraction (see attached figures). In contrast, in both the cytosol fraction and in the mitochondria fraction, these proteins were not detectable.

**ST Cells Plasma Cyto Mito**

**U87 memb**


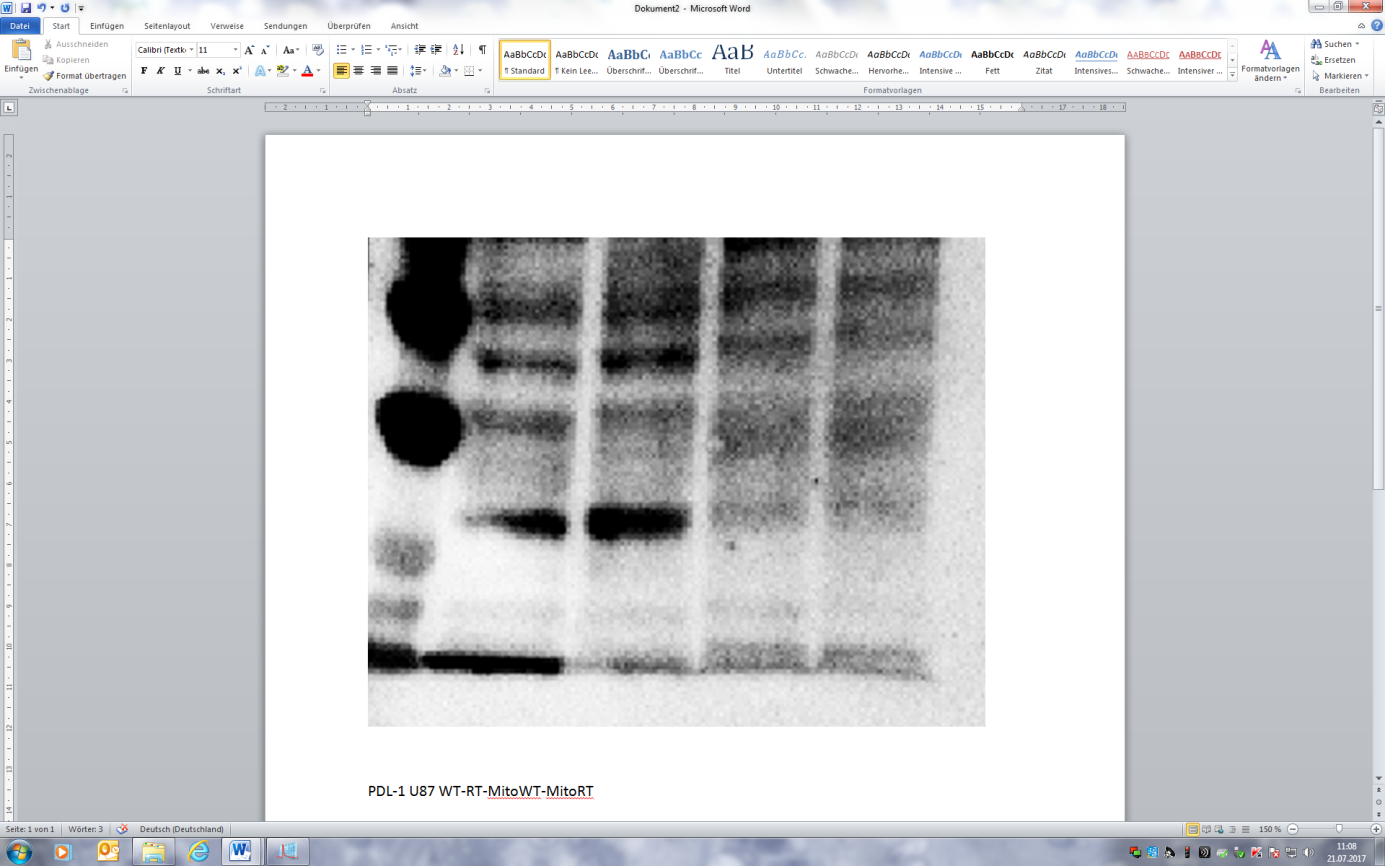


**PD-L1**

**ST Cells Plasma Cyto Mito**

**U87 memb**


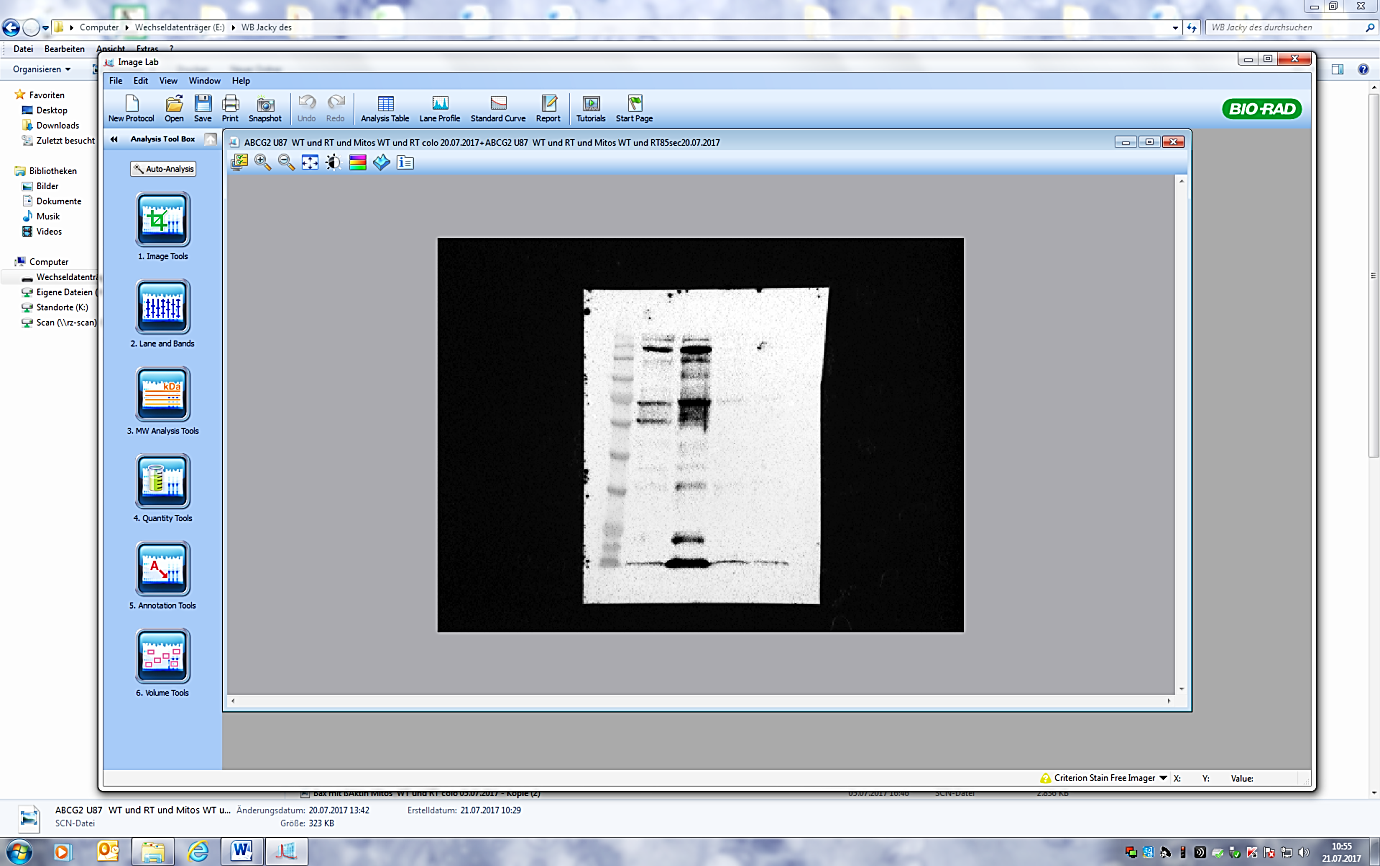


**ABCG2**


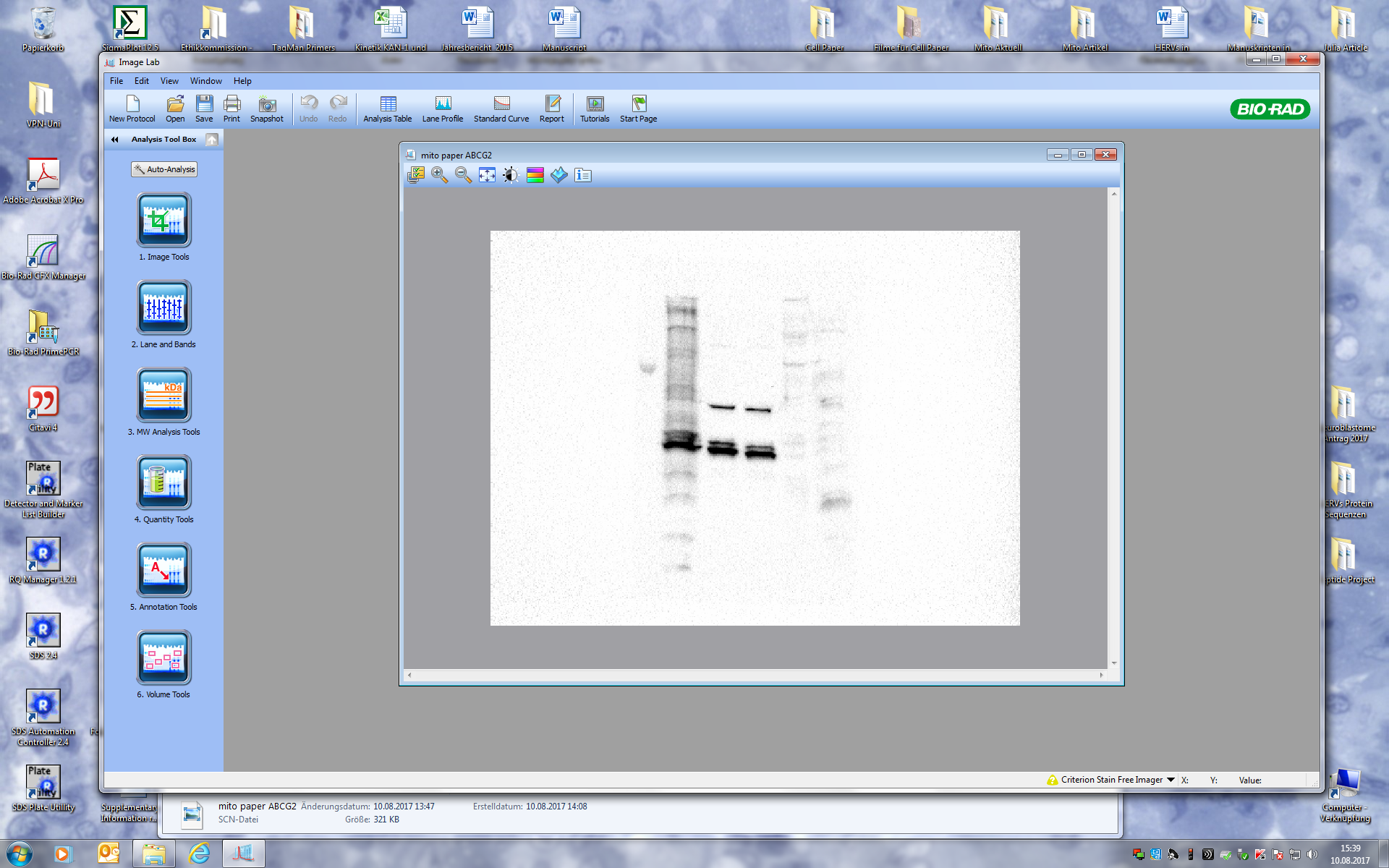


**Cells Nuclear Plasma Mito Cytosol**

**U87 memb**

**ABCG2**

**Legend**:

ST: Standards

Cells U87: Glioblastoma cell line

Nuclear: Nuclear fraction (crude)

Plasma memb: Cell membrane from U87 cells

Cyto: Cytosol

M^RT^G+B: Mitochondrial fraction isolated via sucrose gradient with additional re-purification using magnetic beads.

ABCG2: is a member of the ATP-binding cassette transporter family and a cell membrane protein with six transmembrane spanning regions. The detecting antibody used was purchased from Cell Signaling Cat. # 4477. Expected protein at MW 65-75 KDa (two bands).

PD-L1: The programmed death-ligand 1 (PD-L1) is a 40 kDa type 1 transmembrane protein localized in the cell membrane. The antibody used was purchased from Biorbyt Cat. # orb 10162. Expected protein at MW 32 KDa.

We performed additional experiments to answer this question. BAX is reported to be transported to mitochondria from cytoplasm after apoptotic stimuli. In general there is little basal expression of BAX in all cell cultures, since there is always an existence of cells undergoing apoptosis. We therefore analyzed in the mitochondrial fraction the distribution of BAX in both untreated versus treated U87 cells in the revised manuscript. The same analysis was performed for CoxIV. Furthermore, we compared the distribution of BAX (**A**) and CoxIV (**B**) in mitochondria treated vs. untreated using either sucrose gradients alone or a combination of sucrose gradient and anti-TOM_22_ MicroBeads:


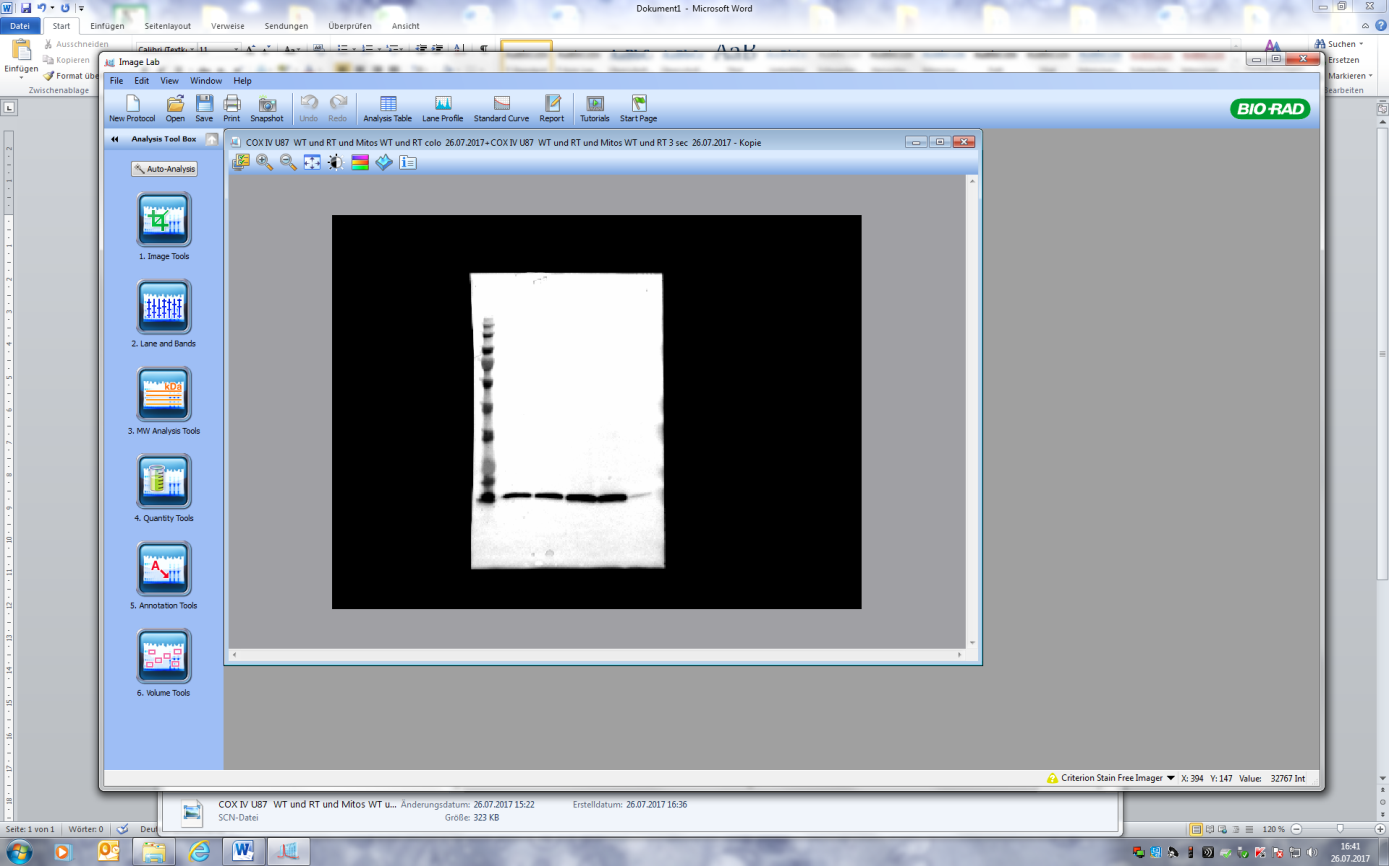


**Cox IV**

**untreated treated untreated treated**

**ST gradient gradient gradient + gradient +**

**anti-TOM MB anti-TOM MB**


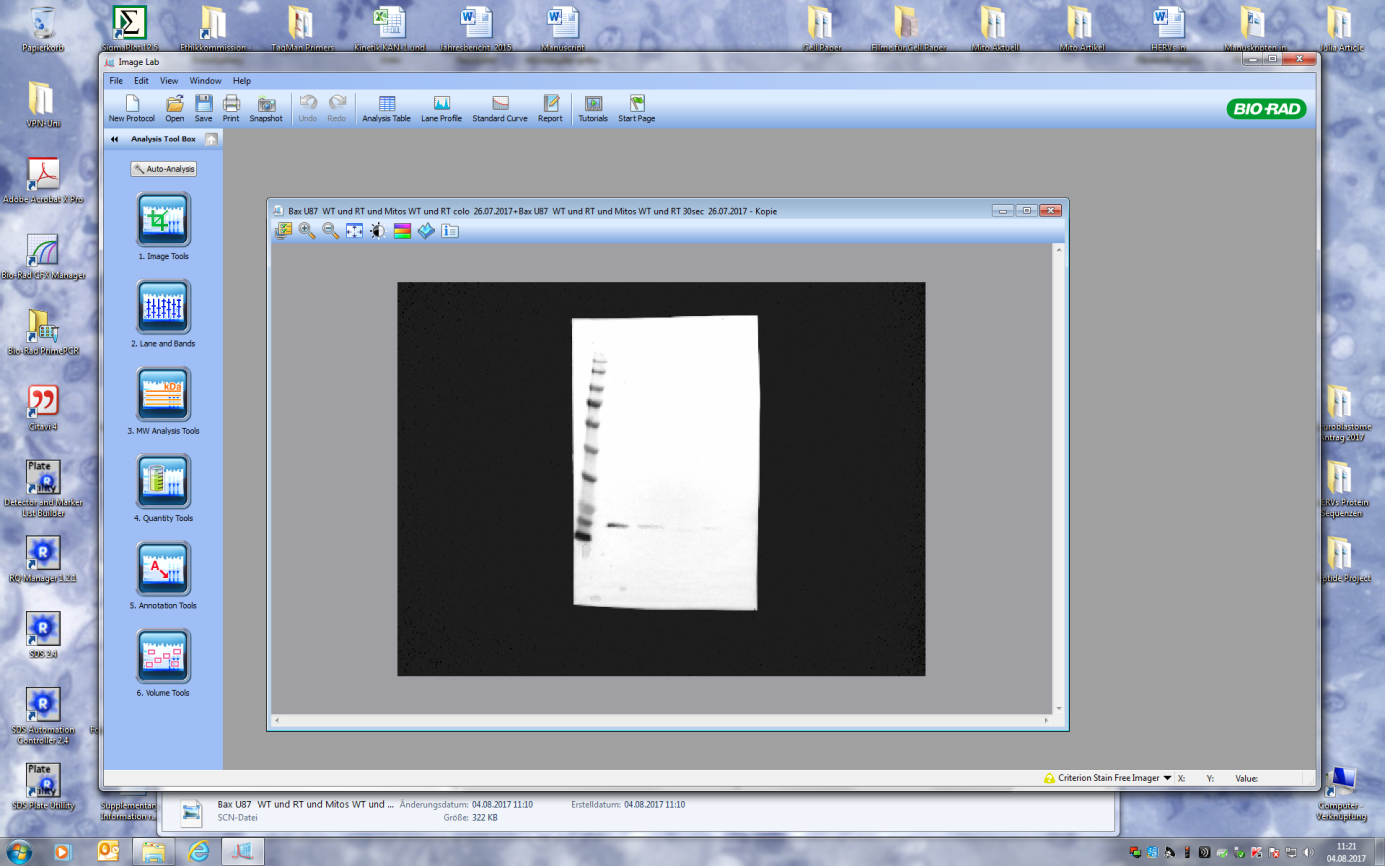


**BAX**

**untreated treated untreated treated**

**ST gradient gradient gradient + gradient +**

**anti-TOM MB anti-TOM MB**

**A**

**B**

Regarding CoxIV protein amounts, we can clearly show no difference between treated vs. untreated cells in the mitochondrial fraction using both systems. Regarding BAX, we could see only a faint signal in mitochondria isolated from untreated cells, in treated cells this signal even appears weaker. In mitochondria isolated via TOM_22_-magnetic-beads separation, we could not see any signal at all. Therefore we conclude that U87 cells after treatment with etoposide do not show any significant signals of apoptosis.

**A**: Comparison of HERV-FRD_1_ protein localization in the mitochondrial fraction isolated by sucrose gradient alone or the combination of sucrose gradient plus anti-TOM_22_ magnetic beads. Results are very similar. **B**: Detection of HERV-FRD_1_ protein in mitochondria isolated using the dual system sucrose gradient and anti-TOM_22_ magnetic beads. Syncytins were detected in whole cell extracts, cell membrane and mitochondrial fraction. In the cytosol the detection of this viral protein was absent. Therefore we can be confident that syncytins are translocalized to the mitochondria.


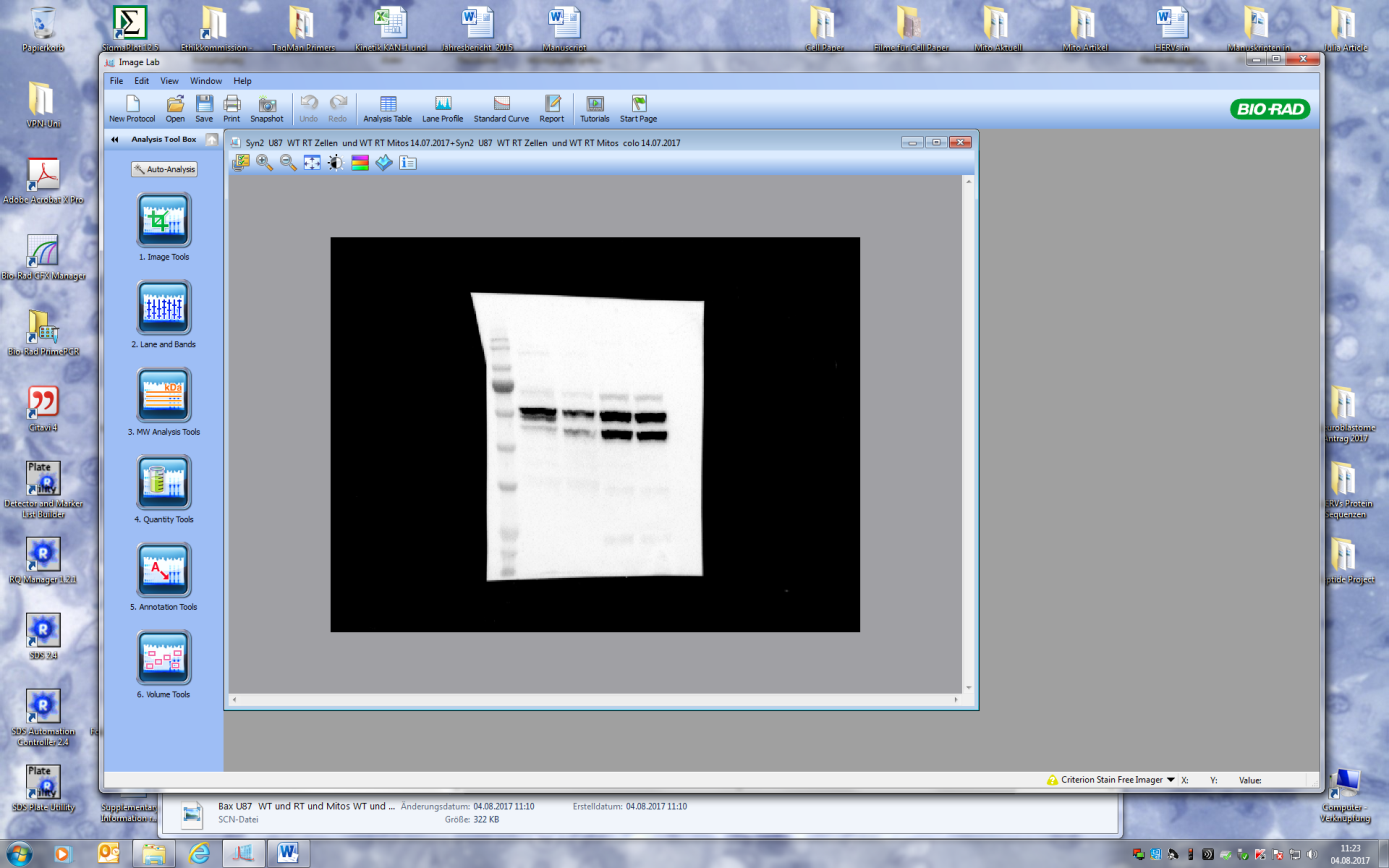


**HERV-FRD_1_**

**untreated untreated treated treated**

**ST gradient gradient + gradient gradient +**

**anti-TOM MB anti-TOM MB**

**HERV-FRD_1_**

Cells Cell memb Mitochon Cytosol


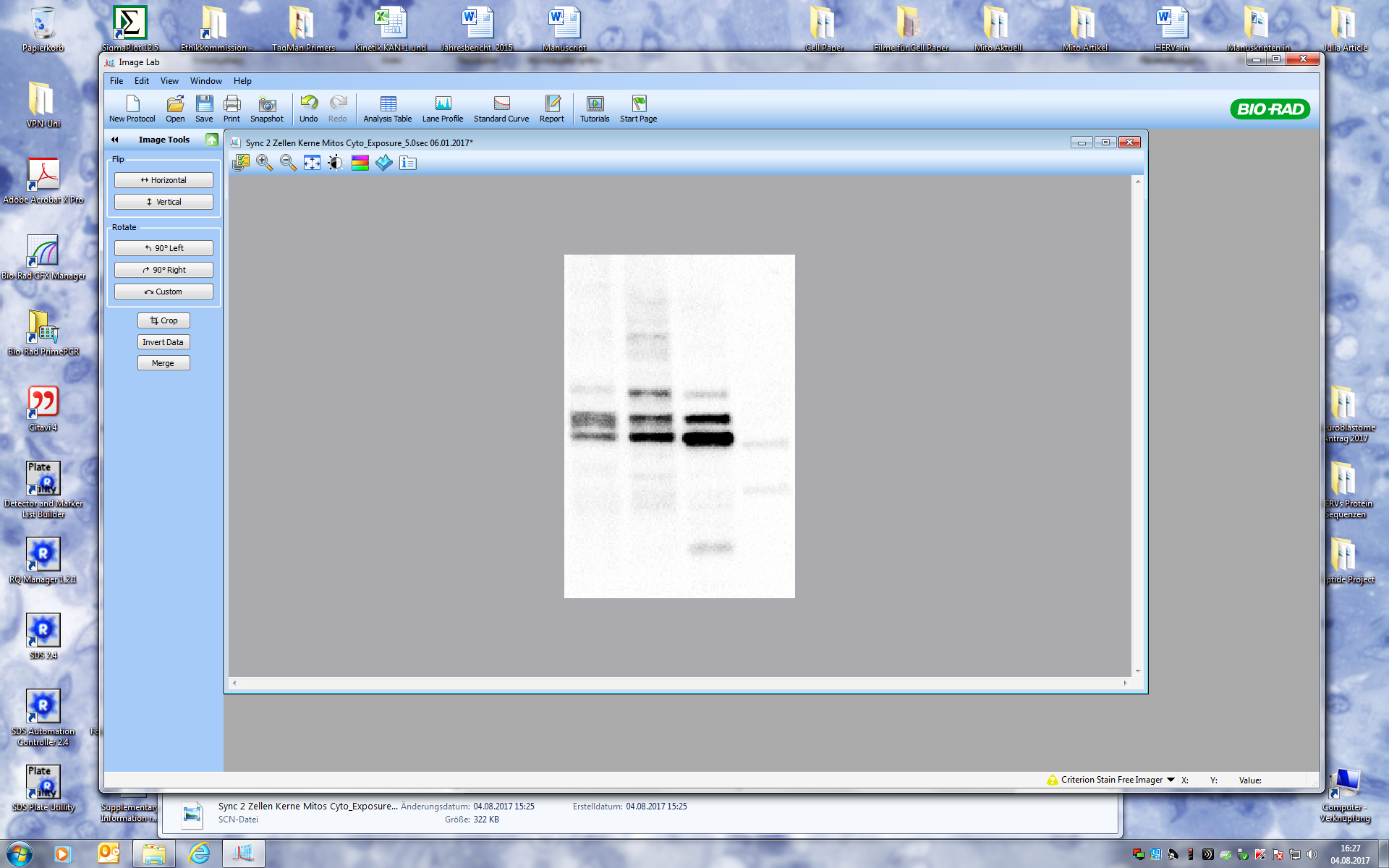


**A**

**B**
